# Charge Transfer Mechanism on a Cobalt-Polyoxometalate-TiO_2_ Photoanode for Water Oxidation in Acid

**DOI:** 10.1021/jacs.4c01441

**Published:** 2024-05-15

**Authors:** Fengyi Zhao, Ting Cheng, Xinlin Lu, Nandan Ghorai, Yiwei Yang, Yurii V. Geletii, Djamaladdin G. Musaev, Craig L. Hill, Tianquan Lian

**Affiliations:** † Department of Chemistry, Emory University, Atlanta, Georgia 30322, United States; ‡ Cherry L. Emerson Centre for Scientific Computation, Emory University, 1515 Dickey Drive, Atlanta, Georgia 30322, United States

## Abstract

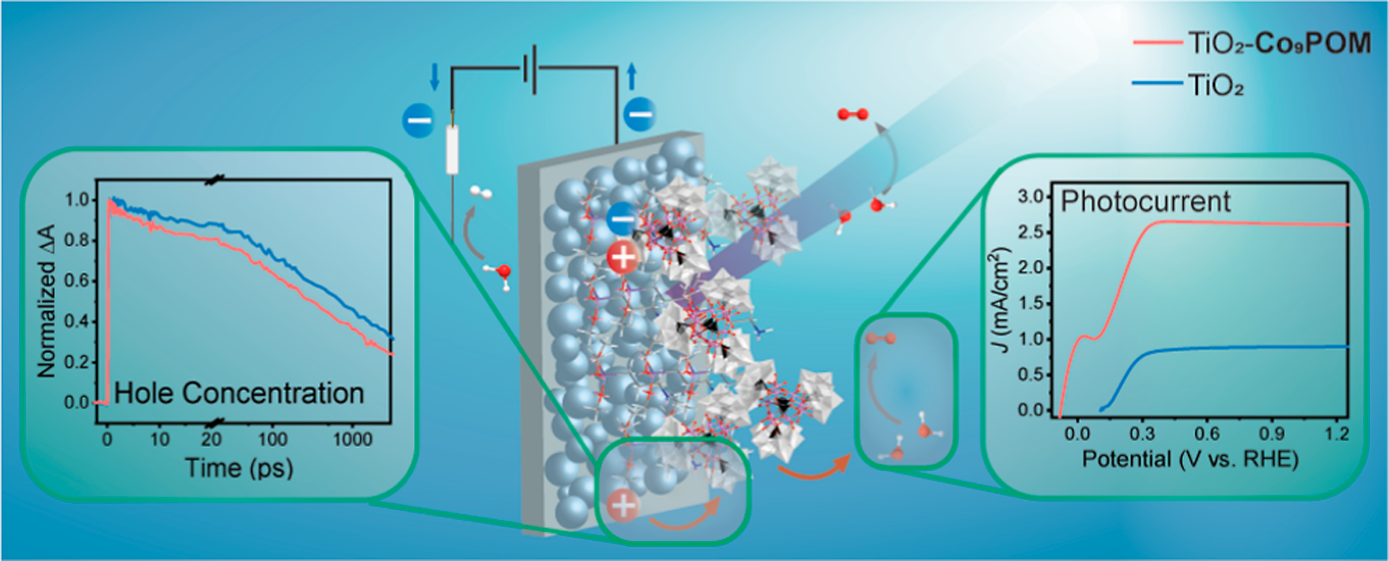

We constructed a photoanode comprising the homogeneous water oxidation catalyst (WOC) Na_8_K_8_[Co_9_(H_2_O)_6_(OH)_3_(HPO_4_)_2_(PW_9_O_34_)_3_] (**Co**_**9**_**POM**) and nanoporous *n*-type TiO_2_ photoelectrodes (henceforth “TiO_2_–**Co**_**9**_**POM**”) by first anchoring the cationic 3-aminopropyltrimethoxysilane (APS) ligand on a metal oxide light absorber, followed by treatment of the metal oxide-APS with a solution of the polyoxometalate WOC. The resulting TiO_2_–**Co**_**9**_**POM** photoelectrode exhibits a 3-fold oxygen evolution photocurrent enhancement compared to bare TiO_2_ in aqueous acidic conditions. Three-element (Co 2p, W 4f, and O 1s) X-ray photoelectron spectroscopy and Raman spectroscopy studies before and after use indicate that surface-bound **Co**_**9**_**POM** retains its structural integrity throughout all photoelectrochemical water oxidation studies reported here. Extensive charge-transfer mechanistic studies by photoelectrochemical techniques and transient absorption spectroscopy elucidate that **Co**_**9**_**POM** serves as an efficient WOC, extracting photogenerated holes from TiO_2_ on the picosecond time scale. This is the first comprehensive mechanistic investigation elucidating the roles of polyoxometalates in POM-photoelectrode hybrid oxygen evolution reaction systems.

## Introduction

The generation of efficient and technologically viable alternative fuel systems via solar energy conversion continues to be the focus of many capable scientists and engineers.^1−5^ The integral part of the currently envisioned solar and photoelectrochemical fuel systems is the formulation of robust catalytic photoanodes consisting of semiconductors and water oxidation catalysts (WOCs)^6−8^ capable of driving the catalytic oxygen evolution reaction (OER). Ongoing extensive research has made significant progress in developing electrocatalytic OER systems in acid that contain a variety of WOCs, such as iridium single-atoms,^9^ RuO_2_,^10^ MnO_*x*_,^11,12^ MoS_2_,^13^ and cobalt-based mixed metal oxides.^13−18^ To date, several photoelectrode hybrid systems consist of semiconductors as light absorbers and WOCs as cocatalysts, including MnO_*x*_,^19^ IrO_*x*_,^20−23^ (NiFe)O_*x*_,^24−26^ Fe(Ni)OOH,^27,28^ Co–Pi,^29−36^ and polyoxometalates (POMs)^37−39^ have been formulated. However, the exact function(s) of the WOC cocatalysts on the photoelectrodes, nature of the utilized electrode materials, stability of these catalytic photoanodes, and the photophysical dynamics at the semiconductor/WOC junction remain the subject of extensive debate. For example, transient absorption (TA) spectroscopy studies have shown that modification of Co–Pi predominantly increases band bending and suppresses electron–hole recombination in Fe_2_O_3_ and BiVO_4_ photoanodes rather than serving as active WOC sites.^30,31,36^ On the other hand, the studies by impedance spectroscopy studies^35^ and potential sensing techniques^29^ have demonstrated that Co–Pi acts as both a hole collector and an OER catalyst on metal oxide photoanodes. Clearly, additional investigations are needed to understand how cocatalysts bound to semiconductor photoanode surfaces and enhance OER performance.

In these circumstances, the catalytic photoanodes comprising a homogeneous WOC supported on a solid electrode offer the possibility of controllable photoanodes by tuning both the metal oxide support (MO_*x*_) and the molecular WOC. Moreover, molecular WOCs have discrete geometrical and electronic structures that aid precise experimental and computational investigations of their multiple photophysical and chemical properties. However, the detailed MO_*x*_–WOC interaction mechanism, especially the role of the WOC on MO_*x*_, is poorly understood.^40,41^ For this purpose, here, we focus on cobalt polyoxometalates as molecular catalysts, which previously were shown to be effective WOCs in both dark and light-driven conditions.^42−53^ Furthermore, previously, POM-WOCs have been successfully immobilized on semiconductors by utilizing multiple methods.^37−39,54,55^ In this study, we select the molecular [Co_9_(H_2_O)_6_(OH)_3_(HPO_4_)_2_(PW_9_O_34_)_3_]^16–^ (**Co**_**9**_**POM**) system as POM-WOC and immobilize it on the well-studied TiO_2_ photoanode. Previous studies showed that the barium salt of **Co**_**9**_**POM**(47) in a carbon paste anode is a more effective WOC than the operationally optimal heterogeneous WOCs IrO_2_ and Co_3_O_4_.^45^

Briefly, we found that the resulting TiO_2_–**Co**_**9**_**POM** photoanode is robust in acid and exhibits enhanced current densities compared to those of bare TiO_2_ and other control TiO_2_ modifications. Several photoelectrochemical techniques and TA spectroscopy in two different spectral regions enable us to probe the interfacial charge transfer process from the photoanode to the **Co**_**9**_**POM** WOC and to identify key charge-transfer mechanistic insights in this TiO_2_–**Co**_**9**_**POM** photoanode system.

## Results and Discussion

### Characterization of TiO_2_ and TiO_2_–**Co**_**9**_**POM** Photoelectrodes

**Figure 1:**
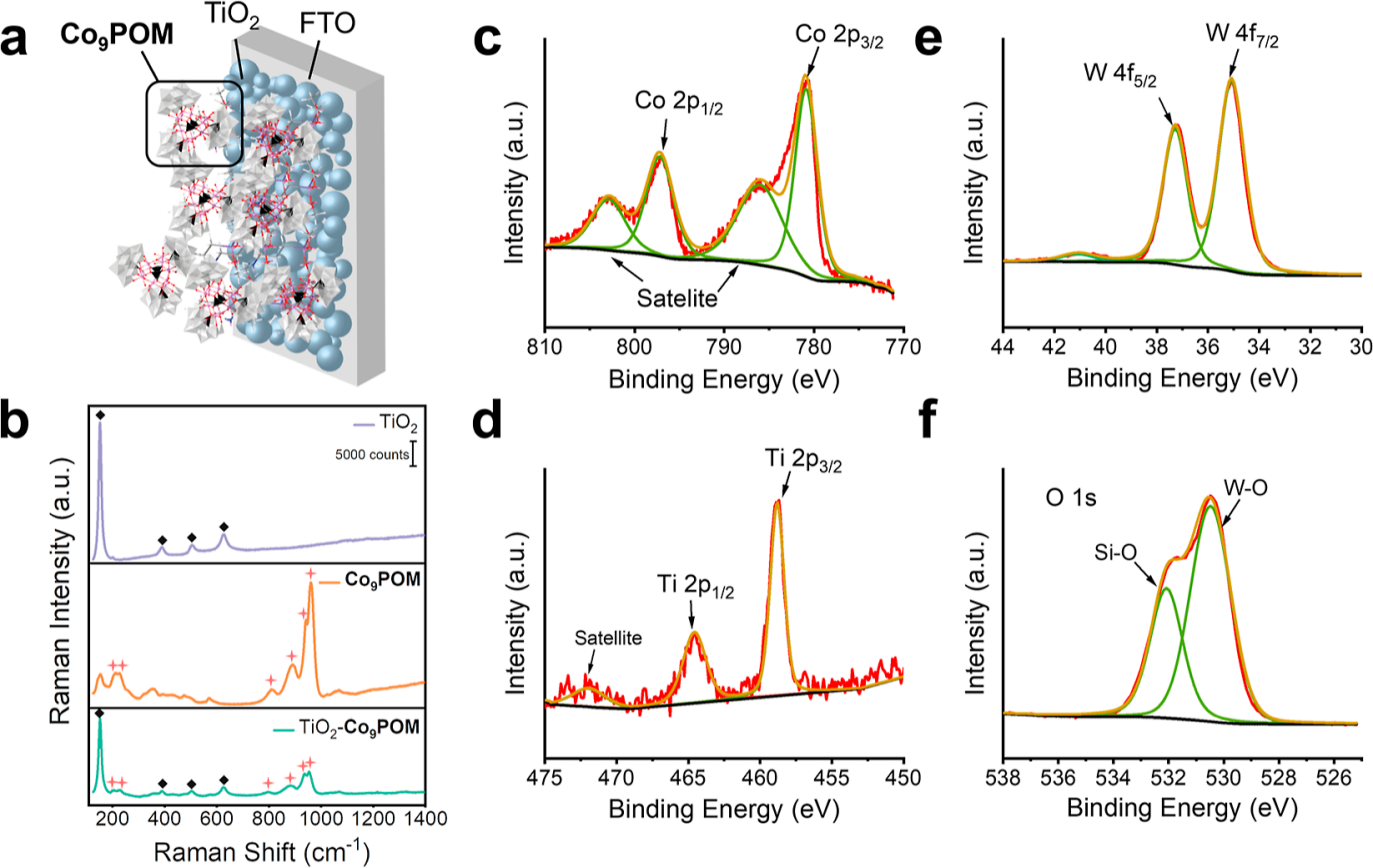
(a) Schematic illustration of the TiO_2_–**Co**_**9**_**POM** hybrid electrode. (b) Ex situ Raman spectroscopy of TiO_2_, solid **Co**_**9**_**POM**, and TiO_2_–**Co**_**9**_**POM** on FTO substrates, where diamonds and stars represent the characteristic Raman peaks of anatase TiO_2_ and **Co**_**9**_**POM**, respectively. (c–f) Co 2p, Ti 2p, W 4f, and O 1s are the X-ray photoelectron spectra of the as-prepared TiO_2_–**Co**_**9**_**POM** sample.

**Co**_**9**_**POM** and **Co**_**9**_**POM**-modified TiO_2_ photoelectrodes were synthesized and purified, as described in the materials section of the Supporting Information. Briefly, after treating nanoporous TiO_2_ films with the cationic silylating agent 3-aminopropyltrimethoxysilane (APS), the anionic **Co**_**9**_**POM** absorbs onto the cationic TiO_2_–APS substrate to form three component TiO_2_–APS–**Co**_**9**_**POM** photoanodes (referred to henceforth as “TiO_2_–**Co**_**9**_**POM**” for brevity). Repeated washings of the TiO_2_–**Co**_**9**_**POM** with organic solvents as well as water show no displacement of the POM into solution, consistent with the strong electrostatic binding of **Co**_**9**_**POM** (16-charge) and the positively charged terminal ammonium groups of the APS units as well as the robust covalent bonding of the silyl terminus of APS with the TiO_2_ surface oxygens, as shown in Figure 1a. Several techniques indicate that intact **Co**_**9**_**POM** is successfully immobilized on the TiO_2_ surface. First, Raman spectroscopy shows the attachment of **Co**_**9**_**POM** to TiO_2_, as shown in Figure 1b. TiO_2_ displays four characteristic peaks (black diamonds) of the anatase phase at 151, 390, 505, and 626 cm^–1^, corresponding to the *E*_g_, *B*_1g_, *A*_1g_, and *E*_g_ modes, respectively.^56,57^ Second, the Fourier transform infrared spectrum (Figure S1a) of solid **Co**_**9**_**POM** shows stretching peaks, which are characteristic of Keggin-structure-derived polytungstates at 803 cm^–1^ ν(W–O_c_–W, c = octahedral edge-sharing), 886 cm^–1^ ν(W–O_b_–W, b = octahedral corner-sharing), 935 cm^–1^ ν(W–O_d_, d = terminal), and 1029 cm^–1^ ν(P–O_a_, a = tetrahedral). These peaks are consistent with previously reported **Co**_**9**_**POM**.^45^ The well-documented vibrational peaks of **Co**_**9**_**POM** are also observed in the Raman spectrum of TiO_2_–**Co**_**9**_**POM** (red stars, Figure 1b).^45^ On TiO_2_–**Co**_**9**_**POM**, the characteristic peaks of both TiO_2_ and **Co**_**9**_**POM** are clearly present, confirming successful surface immobilization of **Co**_**9**_**POM** on TiO_2_.

Third, the X-ray photoelectron spectroscopies (XPS) conducted on the TiO_2_–**Co**_**9**_**POM** film clearly show the presence of TiO_2_ (Ti 2p peak and O 1s peak), APS (C 1s peak, N 1s peak, and Si 2p peak), and **Co**_**9**_**POM** (Co 2p peak, W 4f peak, and O 1s peak), as shown in Figures 1c–f and S2. The XPS spectrum of Ti 2p (Figure 1d) has significantly split spin–orbit components (Δ_oxide_ = 5.7 eV) with the Ti 2p_3/2_ peak and the Ti 2p_1/2_ peak located at 458.5 and 464.2 eV, respectively. A broad satellite peak at 472 eV is consistent with the literature.^58^ The signal-to-noise ratios of these Ti peaks are understandably low, because the TiO_2_ surface is covered by APS and **Co**_**9**_**POM**. The XPS spectrum of W 4f (Figure 1e) shows binding energies of 35.28 eV (4f_7/2_) and 37.48 eV (4f_5/2_), which are typical for W(VI) centers. The loss feature for W(VI) at 41 eV is also observed. Figure 1c displays the XPS spectrum of Co 2p with binding energies of 780.7 eV (2p_3/2_) and 797.1 eV (2p_1/2_). Associated with these peaks are two observable satellite peaks at 786.4 and 803.0 eV. These features correspond to the Co(II) oxidation state and will be used for comparison in the following characterizations. The O 1s XPS spectrum in Figure 1f can be fitted with two peaks located at 530.5 and 532.2 eV, which are assigned to oxygen atoms in the chemical environment of W–O (POM) and Si–O (APS), respectively. ICP experiments quantify a Co loading of 7.7 μg per cm^2^ of sample geometric area, corresponding to ca. 0.9 wt % of Co on TiO_2_.

### Photoelectrochemical OER Performance of TiO_2_–**Co**_**9**_**POM**

**Figure 2:**
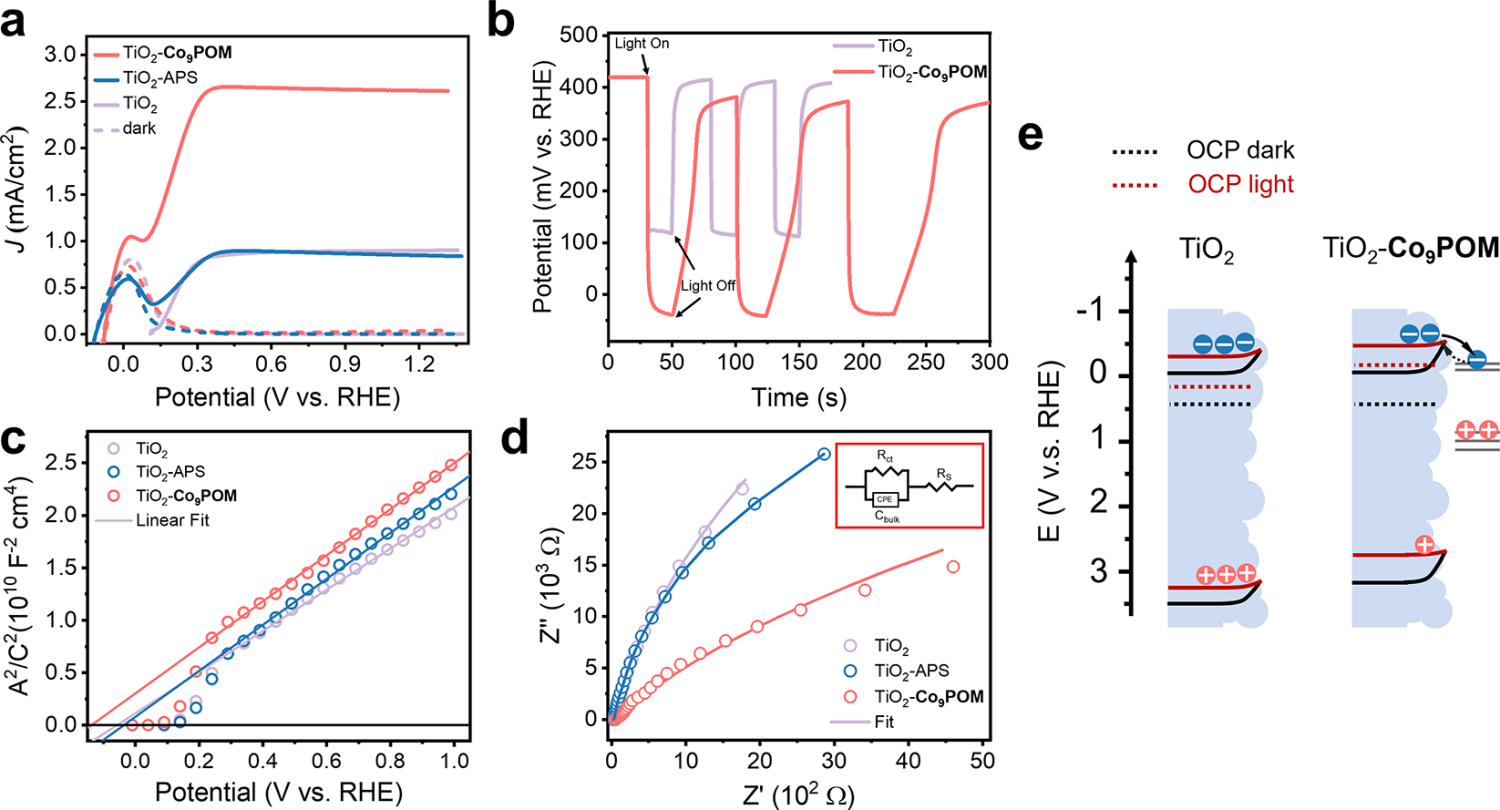
Photoelectrochemical characterization of TiO_2_–**Co**_**9**_**POM**. (a) *J*–*V* curve of TiO_2_, TiO_2_–APS, and TiO_2_–**Co**_**9**_**POM** in the dark (dash curve) and under 100 mW/cm^2^ 365 nm UV illumination (solid curve). (b) OCP of TiO_2_ and TiO_2_–**Co**_**9**_**POM** under dark and 100 mW/cm^2^ 365 nm UV illumination. (c) Mott–Schottky analysis of the TiO_2_, TiO_2_–APS, and TiO_2_–**Co**_**9**_**POM** photoanode under dark conditions. (d) Nyquist plot for electrochemical impedance spectroscopy conducted on TiO_2_ and TiO_2_–**Co**_**9**_**POM** at 1.23 V under 12.8 mW/cm^2^ UV illumination. Inset shows the equivalent circuit to fit the plot. (e) Band structure schematic of TiO_2_ and TiO_2_–**Co**_**9**_**POM** under light and dark OCP process, showing the TiO_2_ conduction band and valence band edge (solid line) and Fermi-level (dashed line) under the dark (black lines) and light (red lines) OCP conditions, and the relevant **Co**_**9**_**POM** electron accepting (oxidation/reduction of W) or hole accepting (oxidation/reduction of Co) levels. All experiments are performed in pH 2 sulfate buffer solution.

The effects of **Co**_**9**_**POM** and APS modification on the TiO_2_ photoelectrode were evaluated in a series of photoelectrochemical (PEC) characterizations in a pH 2 sulfate buffer solution (details in the Experimental Section). Figure 2a shows that under the dark conditions, TiO_2_, TiO_2_–APS, and TiO_2_–**Co**_**9**_**POM** electrodes exhibit negligible current when scanned to positive potentials, though there is an oxidation peak at around 0.0 V vs reversible hydrogen electrode (RHE) that can be attributed to the Ti(III)/Ti(IV) redox couple.^59^ All potentials in this study are referenced to RHE unless otherwise mentioned. With 365 nm light illumination, the photocurrent increases substantially and reaches a plateau at around 0.30 V for TiO_2_, TiO_2_–APS, and TiO_2_–**Co**_**9**_**POM**. Photocurrents on TiO_2_ and TiO_2_–APS are nearly the same, but the saturated photocurrent of TiO_2_–**Co**_**9**_**POM** is nearly three times that of TiO_2_ and TiO_2_–APS.

To uncover the photocurrent enhancement mechanism, a series of photoelectrochemical studies were conducted. Figure 2b shows the open circuit measurement with and without light illumination. The open circuit potentials (OCPs) of TiO_2_ and TiO_2_–**Co**_**9**_**POM** are similar under dark conditions at ∼420 mV. Under light illumination, however, the OCP of TiO_2_–**Co**_**9**_**POM** reaches a more negative value (−0.038 V) than that of TiO_2_ (0.121 V). Moreover, the OCP of TiO_2_–**Co**_**9**_**POM** shows a slower recovery to its dark equilibrium potential when the light is off compared to pure TiO_2_. These phenomena can be attributed to the synergic effect of band edge modification and the charge transfer effect. The first effect can be confirmed by the ca. 100 mV more negative flat-band potential (*U*_fb_) of TiO_2_–**Co**_**9**_**POM** (−0.16 V) compared to TiO_2_ (−0.08 V) and TiO_2_–APS (−0.06 V) extracted from Mott–Schottky analysis (Figure 2c). Furthermore, under dark OCP conditions, where the electrode’s Fermi-level equilibrates with the solution redox species, the OCP of TiO_2_–**Co**_**9**_**POM** and TiO_2_ are nearly identical. This means there is at least 100 mV of built-in potential enhancement in TiO_2_–**Co**_**9**_**POM** compared to TiO_2_ under a certain applied potential. This band edge modification phenomenon arises from the highly charged **Co**_**9**_**POM** creating an interfacial dipole that shifts the band edge to a more negative potential.

Notably, the impedance-potential behavior of the nanoporous TiO_2_ electrode is well described by the Mott–Schottky equation indicates that the TiO_2_ photoanode is not fully depleted by the solution redox species, allowing the presence of band bending in the electrode. Although the size of the TiO_2_ precursor particle is tens of nanometers, the annealing process during electrode preparation sinters the particles together to form interconnected domains of larger sizes that can support band bending. The increased electrode band bending enables a faster electron–hole separation and a larger electron concentration buildup under illumination, as indicated by the OCP_light_. On the other hand, **Co**_**9**_**POM** can extract and store photogenerated holes acting as an efficient WOC, which can also result in a higher electron concentration under light illumination. When illumination is turned off, the OCP on TiO_2_–**Co**_**9**_**POM** relaxes more slowly to the initial dark level of the OCP_dark_ compared to pure TiO_2_. This observation is different from the previous OCP study on phosphorus-modified BiVO_4_, where modified BiVO_4_ exhibits a larger OCP photovoltage and a faster OCP_light_ relaxation when illumination is off.^60^ This can be understood by the more energetically positive electron trap state position on TiO_2_ compared to BiVO_4_, which leads to more rapid electron concentration equilibrium on TiO_2_. Upon surface modification with **Co**_**9**_**POM**, however, POM states with energies close to the TiO_2_ conduction band edge are introduced into the system. These states likely involve the reduction and/or oxidation of tungsten (Figure S5a). These coupled states act as shallow electron trap states and lead to slower attainment of the OCP equilibrium when illumination is ceased, as shown in Figure 2e.

AC impedance measurements performed on TiO_2_, TiO_2_–APS, and TiO_2_–**Co**_**9**_**POM** further reveal the function of APS and the WOC, **Co**_**9**_**POM**, under light illumination. A simple equivalent circuit shown in Figure 2d inset has been adopted to fit the impedance data, where the constant phase element and *R*_ct_ are assigned to the capacitance and the resistance of space charge region, respectively; *R*_s_ is assigned to the resistance arising from the back contact, electrode circuit, and solution. As shown in Figure 2d and Table S1, TiO_2_–**Co**_**9**_**POM** exhibits a smaller trapping resistance and a larger capacitance compared to those of TiO_2_ and TiO_2_–APS. The decreased resistance can be attributed to accelerated hole transfer from TiO_2_ to **Co**_**9**_**POM** and from **Co**_**9**_**POM** to water. Meanwhile, **Co**_**9**_**POM** can store more photogenerated holes as a WOC, which explains the increased photoanode capacitance compared to that of TiO_2_. The impedances of TiO_2_ and TiO_2_–APS samples, however, are similar. These experiments along with the LSV performance and Mott–Schottky experiments indicate that the APS ligands behave more as spectators than as passivation layers on TiO_2_.

**Figure 3:**
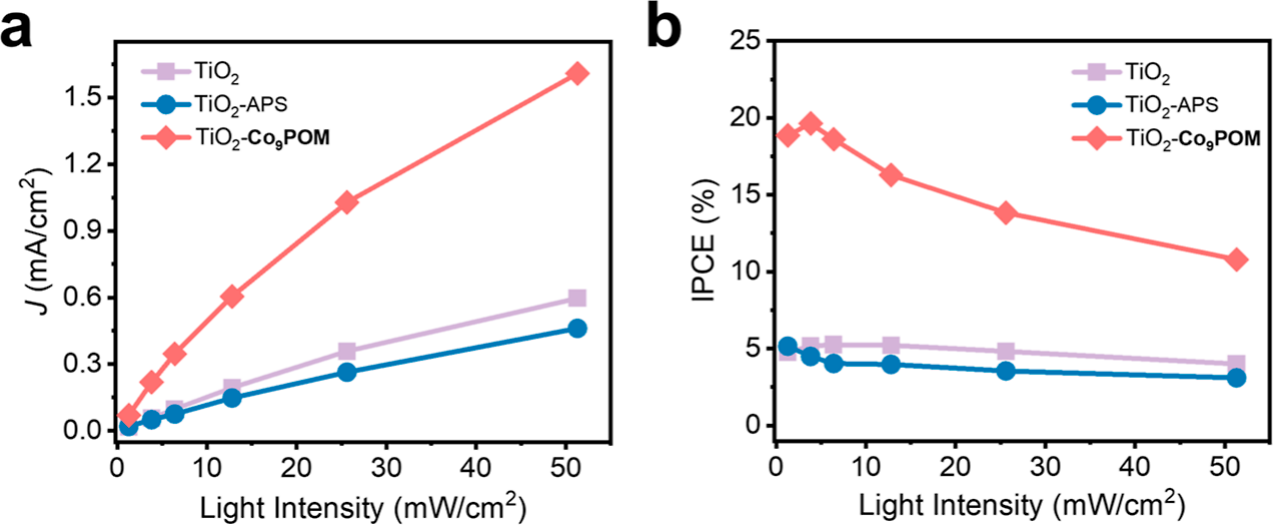
Excitation fluence-dependent (a) photocurrent and (b) incident photon-to-current efficiency (IPCE) of TiO_2_, TiO_2_–APS, and TiO_2_–**Co**_**9**_**POM** measured at 1.23 V under 365 nm UV illumination.

Figure 3a compares the excitation fluence-dependent photocurrent densities of TiO_2_, TiO_2_–APS, and TiO_2_–**Co**_**9**_**POM** photoanodes. The corresponding incident photon-to-current efficiencies (IPCE) are shown in Figure 3b. TiO_2_ and TiO_2_–APS show a steady IPCE of around 5% independent of illumination intensity. The loss of efficiency is mainly attributed to bulk and surface recombination due to the sluggish OER. Notably, on TiO_2_–**Co**_**9**_**POM**, IPCE remains nearly unchanged at around 18% before 6.4 mW/cm^2^ and decreases at higher intensity. This indicates that the catalyst turnover rate lags behind the surface photogenerated hole collection rate at higher illumination intensity, shifting the efficiency-limiting step from photon generation and charge separation to catalyst turnover frequency. This phenomenon was also explored in a prior study on hematite modified with a dinuclear heterogeneous Ir catalyst serving as WOC. The study discovered that the catalyst water oxidation rate constant plateaus when illumination photon flux reaches a certain value due to the increasing hole density on the surface, which leads to decreased IPCE.^21^ These data also indicate that **Co**_**9**_**POM** functions as the catalytic active site in this composite photoanode. It should be pointed out that **Co**_**9**_**POM** has already been demonstrated to exhibit faster WOC kinetics than the state-of the-art heterogeneous WOCs including IrO_2_ and Co_3_O_4_ previously.^45^ It would be very interesting to extend this photon-flux-dependent study to faster catalysts in future work.

**Figure 4:**
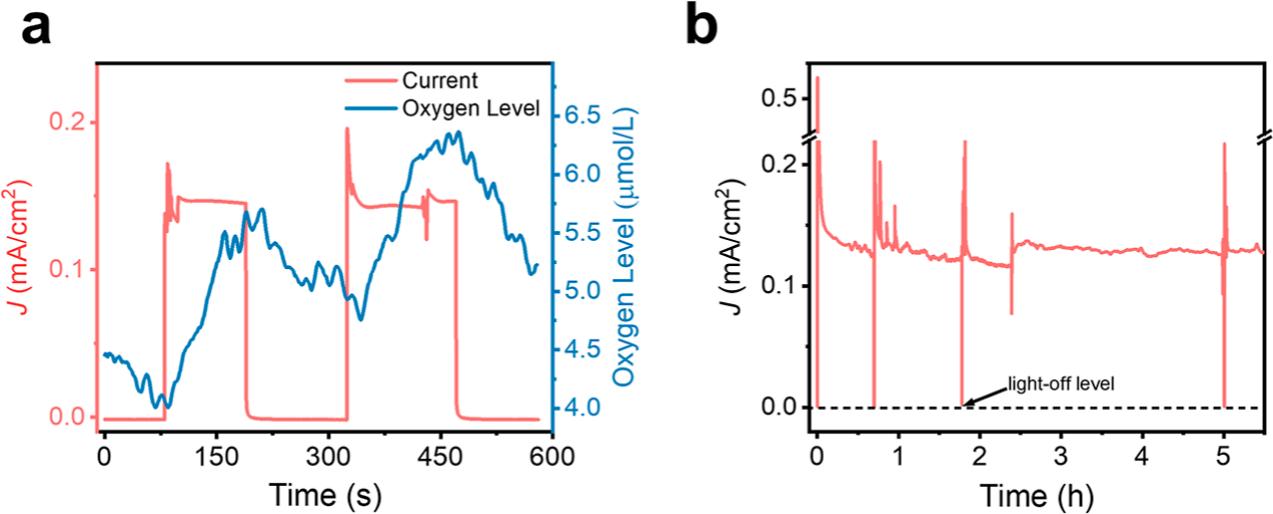
(a) FOXY Forspor oxygen probe during chronoamperometry and (b) chronoamperometric stability test of the TiO_2_–**Co**_**9**_**POM** photoanode collected at 0.73 V under 20 mW/cm^2^ 365 nm UV illumination in pH 2 sulfate buffer.

The formation of oxygen in our system was confirmed by following the response of the oxygen sensor in a chronoamperometry experiment at 0.73 V applied potential and 20 mW/cm^2^ 365 nm UV illumination, as shown in Figures 4a and S6. With the light on, the O_2_ concentration near the electrode increases from 4.0 to 5.5 μM; with the light off, [O_2_] slowly decreases due to diffusion of the O_2_ from the electrode surface to the bulk solution. When the light is on again, [O_2_] increases again from 5.0 to 6.4 μM. These probed concentrations are much lower than the O_2_ solubility in fresh water under 1 atm O_2_, 25 °C (1.2 mM).^61^ Therefore, no gas bubbles are expected on the electrode during the short periods of light illumination. The stability of the electrode and accumulated products was also evaluated under the same chronoamperometry conditions. Figure 4b shows that the photocurrent remains stable for 5 h, passing ca. 3.0 C of charge on the electrode. The initial photocurrent spike is related to capacitive charging, a non-Faradaic process, which was addressed in previous studies.^45^

GC measurement of the accumulated oxygen product shows an OER Faradaic efficiency of 96.5 ± 5.5% after corrections for air leakage into the system. Faradaic efficiency is the ratio of holes contributing to the OER to the total number of photogenerated holes crossing the interface. The near-unity Faradaic efficiency means that nearly all transferred holes are used for the water oxidation reaction rather than some other competing hole oxidation reactions. This data does not conflict with the presence of surface recombination observed in Figure 3 at high illumination flux, where the surface recombination will only result in a decreased hole transfer current across the electrode–electrolyte junction.^62^ X-ray photoelectron spectra of Co, W, and oxygen, as well as Raman spectra of TiO_2_–**Co**_**9**_**POM** photoanodes after the chronoamperometric studies shown in Figure 4b were measured to assess the stability of the surface-anchored POM. XPS shows that both the Co and W peaks are well maintained (Figure S2b–d). Significantly, these peaks are quite distinct from the XPS peaks of Co_3_O_4_ (Figure S3). Characteristic Raman peaks of **Co**_**9**_**POM** on TiO_2_–**Co**_**9**_**POM** are still observed after chronoamperometric studies (Figure S4). These data collectively indicate the structural integrity of **Co**_**9**_**POM** after long-time use and establish that TiO_2_–**Co**_**9**_**POM** acts as an efficient and fairly robust OER photoanode in acidic aqueous media.

Control experiments using TiO_2_ photoelectrodes modified by nontransition-metal substituted POMs and Co–Pi confirm the indispensable role of **Co**_**9**_**POM** for the water oxidation reaction under acidic conditions. Figure S5b–d shows that both H_3_PW_12_O_40_- and Co–Pi-decorated TiO_2_ photoelectrodes exhibit inferior activity than a pure TiO_2_ photoelectrode. For H_3_PW_12_O_40_-modified TiO_2_, the lack of metal catalytic center and the blocking of TiO_2_ surface reaction sites leads to a decreased photocurrent (Figure S5b). These results also confirm that a Co center and not the polytungstate ligands are required for catalytic water oxidation. Significantly, although Co–Pi has been extensively reported as a superior water oxidation cocatalyst in neutral and alkaline solutions,^29−32,34−36^ it is not as active as **Co**_**9**_**POM** in acidic conditions (Figure S5c,d). The decreased photocurrent in Figure S5c may be due to the absorption of Co–Pi at 365 nm, leading to an inferior light harvesting efficiency by TiO_2_. Results of the control experiments are summarized in Table 1. To the best of authors’ knowledge, there is little work that reports that achieve successful immobilization of a POM WOC on a photoelectrode other than that published by our group using a silylating agent as the anchoring group.^38,39^ Studies from other groups that couple POM WOCs with semiconductors mostly utilize semiconductors as photocatalysts, where the absence of applied potential could lead to significant charge carrier recombination.^37,54,55^ More importantly, POMs in those studies are mostly unstable during the photocatalytic process and may degrade to other species which act as the true catalyst.^37,54^

**Table 1:** Modification Comparison Summarized from Figures 2a and S5b,d

	performance compared to TiO_2_	conclusion
TiO_2_–**Co**_**9**_**POM**	3-fold enhancement	enhanced OER
TiO_2_–H_3_PW_12_O_40_	significantly lower	lack of catalytic center
TiO_2_–Co–Pi (back illumination)	1.5-fold enhancement	enhanced OER

### TA Spectroscopy Probing Charge Transfer Dynamics in TiO_2_–**Co**_**9**_**POM**

**Figure 5:**
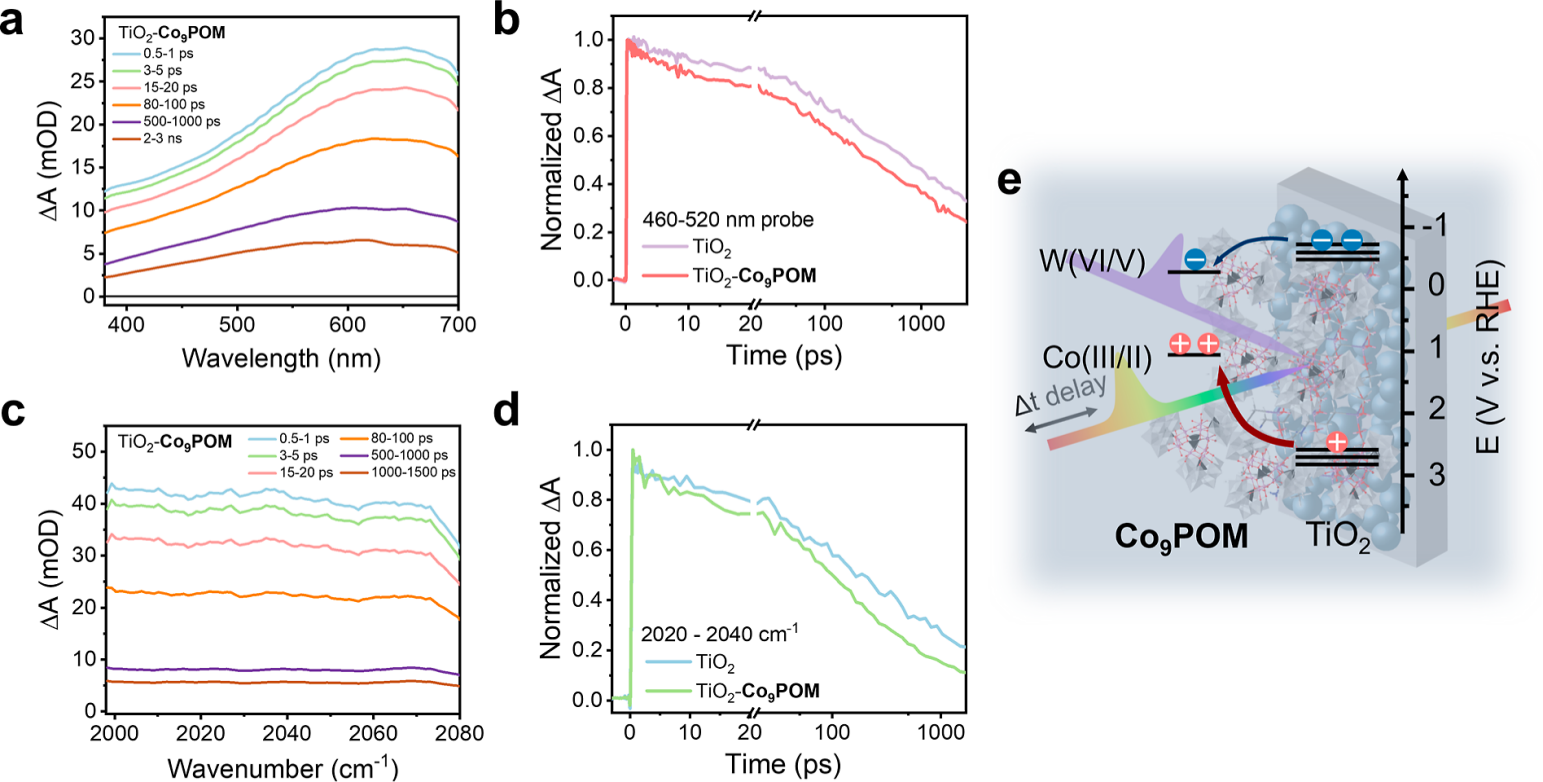
TA spectra of TiO_2_–**Co**_**9**_**POM** films under a 350 nm pump and (a) visible (c) mid-IR probe. Normalized TA decay kinetics on TiO_2_ and TiO_2_–**Co**_**9**_**POM** probed at (b) 460–520 nm in the visible region and (d) 2020–2040 cm^–1^ in the mid-IR following 350 nm excitation. (e) Proposed charge transfer mechanism between TiO_2_ and **Co**_**9**_**POM** upon photoexcitation. Samples for TA-vis experiments are deposited on FTO substrates, samples for TA-IR experiments are deposited on a sapphire window to avoid extensive absorption by tin oxide. An excitation power density of 462 μJ/cm^2^ is used in all TA experiments.

The above PEC studies show that **Co**_**9**_**POM** modification of TiO_2_ strongly enhances the photocurrent, and this can be attributed to **Co**_**9**_**POM** acting as an active OER catalyst. However, from the experiments presented above, it is hard to rule out the possibility that **Co**_**9**_**POM** modification passivates the surface states and suppresses recombination. Therefore, more direct evidence of the charge transfer dynamics is needed. Herein, we apply TA spectroscopies to study the carrier dynamics of TiO_2_, TiO_2_–APS and TiO_2_–**Co**_**9**_**POM** photoanodes in both visible (TA-vis) and mid-IR regions (TA-IR), as shown in Figures 5, S7 and S8. Before detailed analysis of the TA spectra and kinetics of TiO_2_–**Co**_**9**_**POM**, a series of control experiments were done on TiO_2_ to assign the TA spectral features observed in the visible range. As shown in Figures 5a and S7a, similar broad positive signals were observed upon excitation of both TiO_2_ and TiO_2_–**Co**_**9**_**POM** films on the FTO substrate. The broad signal in the visible range is assigned to be contributions of both photogenerated electrons and holes, where the trapped hole and electrons have maximum absorption peaks at 520 and 770 nm, respectively.^63−66^ We extract the TiO_2_ kinetics from 460 to 520 nm and compare their decay behavior in DI H_2_O, an electron scavenger solution (0.1 M Na_2_S_2_O_8_ aqueous solution), and a hole scavenger solution (30% methanol aqueous solution) in Figure S7d. The decay kinetics are much faster in 30% methanol solution and slightly slower in the electron scavenger solution. This indicates that the kinetics from 460 to 520 nm mainly originate from photogenerated holes in nanoporous TiO_2_. Kinetics in this range can be further used to follow the decay of photogenerated holes. Upon **Co**_**9**_**POM** oxidation by holes on TiO_2_, although a broad positive oxidized POM signal may appear in the visible spectrum, such signal, if any, would be negligible (<0.1 mOD) compared to the signal of TiO_2_ hole signal (>10 mOD) mainly resulting from its weak d–d transition.^67^ Therefore, we believe the kinetics from 460 to 520 nm can also track the kinetics of TiO_2_ photogenerated holes on the TiO_2_–**Co**_**9**_**POM** sample.

Excitation fluence-dependent hole kinetic decays are compared in Figure S7b. The initial signal amplitude scales linearly with power density, but the decay half-time and residual signal amplitude at 1 ns decrease at higher excitation power, as shown in Figure S7c. This indicates that the number of photogenerated holes scales linearly with excitation power, but recombination becomes faster with more generated carriers. The fluence-dependent hole kinetics can be fit well by a power law decay *A* = (*t* – *t*_0_)^−β^, with an β of 0.25 ± 0.002 for all decays. This dispersive bimolecular recombination is often observed in metal oxides and organic semiconductor materials.^68−71^ This can be explained by trap-assisted electron–hole recombination. Figures 5b and S7e further compare the hole decay kinetics of the TiO_2_, TiO_2_–APS, and TiO_2_–**Co**_**9**_**POM** photoelectrodes. Hole decay kinetics in TiO_2_ and TiO_2_–APS are similar, while signal decay accelerates in TiO_2_–**Co**_**9**_**POM**. This further clarifies the previous proposals: **Co**_**9**_**POM** acts as a WOC that extracts photogenerated holes on the picosecond time scale rather than suppressing the trap-assisted electron–hole recombination from the picosecond to nanosecond time scale by passivating surface states, while APS acts as a spectator and does not affect the hole kinetics. What needs to be noted is that the hole kinetics observed in Figure 5b account for the hole population of the whole TiO_2_ film (∼40 μm), where most carriers experience bulk recombination. The charge transfer effect from TiO_2_ to **Co**_**9**_**POM** will be more obvious if only the surface hole that can diffuse or drift to the TiO_2_ surface is considered (∼1 μm).^72^

In addition, TA-IR spectroscopies were conducted on TiO_2_, TiO_2_–APS, and TiO_2_–**Co**_**9**_**POM** samples to probe the photogenerated electron kinetics in the TiO_2_ conduction band, as shown in Figures 5c,d and S8. All samples showed a broad positive signal from 2000 to 2080 cm^–1^ upon excitation. The signal can be assigned to the TiO_2_ conduction band-free electron absorption.^73−75^ The TΑ-IR spectra rule out the contribution of **Co**_**9**_**POM** and APS excited states or oxidized states due to the lack of IR transitions in this region (Figure S1a and ref (76)). As shown in Figure S8d, the electron signal in TiO_2_ shows similar decay features with or without APS treatment, meaning that APS is not reduced by the TiO_2_ conduction band electrons. Notably, the electron signal decays faster when **Co**_**9**_**POM** is attached to TiO_2_–APS, which is entirely consistent with TiO_2_ conduction band electrons capable of reducing tungsten in **Co**_**9**_**POM**, a thermodynamically favorable process (∼−0.2 V, Figure S5a, compared to TiO_2_ conduction band edge, −0.4 V).^77^ These findings are also in agreement with the slow recovery of the OCP from light to dark states of TiO_2_–**Co**_**9**_**POM**, where tungsten states close to the TiO_2_ conduction band are introduced in the coupled system. Although TA-IR is conducted on a dry film without any applied potential, it resembles the OCP condition under light illumination, where band bending is largely flattened due to electron accumulation, as depicted in Figure 2e.^78,79^ The band alignment at the OCP_light_ is therefore close to the scenario of an unbiased solid–air interface where little band bending exists. Despite this unfavorable electron transfer process observed in dry film TA-IR measurement and under OCP_light_ conditions, where photogenerated charge carriers barely separate, the unfavorable electron transfer pathway can be greatly mitigated under the OER conditions as sufficient positive applied potential can decrease the electron population in nanoporous TiO_2_. In fact, the fluence-dependent IPCE experiments shown in Figure 3 have demonstrated that the efficiency-limiting factor is mainly the bulk recombination and the insufficient catalyst turnover rate at high illumination power density rather than electron transfer from TiO_2_ to **Co**_**9**_**POM**. In summary, our TA-vis experiments successfully characterize the hole transfer from the TiO_2_ photoanode to the WOC, **Co**_**9**_**POM**. The TA-IR experiments further suggest that TiO_2_–**Co**_**9**_**POM** is a highly coupled system, even with the attachment of an insulting APS layer. There may be two possible roles of APS: one is to act as a tunneling barrier, allowing for electron or hole tunneling from TiO_2_ to WOCs; the other possible role is to serve as a supporting layer to allow direct contact between POM and TiO_2_. It is noteworthy that the microenvironment effect, including the effect introduced by the cationic groups and POM counterions, may have an impact on photocurrent performance.^67^ These effects will be more carefully investigated in our following work.

## Conclusions

We report a POM immobilization method by first anchoring the cationic APS ligand on a metal oxide light absorber, followed by treatment of the metal oxide-APS with a solution of a POM WOC, **Co**_**9**_**POM**. The resulting **Co**_**9**_**POM**-functionalized TiO_2_ photoelectrode, TiO_2_–**Co**_**9**_**POM** in this study, exhibits a 3-fold photocurrent enhancement compared to bare TiO_2_ in aqueous acidic conditions. The structural integrity of the catalyst, **Co**_**9**_**POM**, is maintained for up to 5 h of operation. To the best of our knowledge, this is the first application of an efficient POM-based catalytic photoanode that achieves efficient water photo-oxidation in acid. Extensive mechanistic studies conclude that the enhancement of photocurrent is mainly due to **Co**_**9**_**POM** acting as a fast hole collector and simultaneously as an active catalytic center rather than as a passivator of surface states. TA spectroscopy further verifies the fast photogenerated hole transfer from TiO_2_ to **Co**_**9**_**POM** at the ps time scale. The highly charged **Co**_**9**_**POM**-modified surface also modulates the TiO_2_ band edge, enabling a suitable surface electric field to separate the photogenerated charge carrier.

## Supporting Information Available

Additional experimental methods and control experimental data, including general characterization, photoelectrochemical characterization, and TA spectroscopy characterization (PDF)

## References

[ref1] Lewis N. S.; Nocera D. G. Powering the planet: Chemical challenges in solar energy utilization. Proc. Natl. Acad. Sci. U.S.A. 2006, 103, 15729–15735. 10.1073/pnas.0603395103. 17043226 PMC1635072

[ref2] Hisatomi T.; Kubota J.; Domen K. Recent advances in semiconductors for photocatalytic and photoelectrochemical water splitting. Chem. Soc. Rev. 2014, 43 (22), 7520–7535. 10.1039/C3CS60378D. 24413305

[ref3] Takata T.; Jiang J.; Sakata Y.; Nakabayashi M.; Shibata N.; Nandal V.; Seki K.; Hisatomi T.; Domen K. Photocatalytic water splitting with a quantum efficiency of almost unity. Nature 2020, 581 (7809), 411–414. 10.1038/s41586-020-2278-9. 32461647

[ref4] Zhou P.; Navid I. A.; Ma Y.; Xiao Y.; Wang P.; Ye Z.; Zhou B.; Sun K.; Mi Z. Solar-to-hydrogen efficiency of more than 9% in photocatalytic water splitting. Nature 2023, 613 (7942), 66–70. 10.1038/s41586-022-05399-1. 36600066

[ref5] Stamenkovic V. R.; Strmcnik D.; Lopes P. P.; Markovic N. M. Energy and fuels from electrochemical interfaces. Nat. Mater. 2017, 16 (1), 57–69. 10.1038/nmat4738. 27994237

[ref6] Han L.; Dong S.; Wang E. Transition-Metal (Co, Ni, and Fe)-Based Electrocatalysts for the Water Oxidation Reaction. Adv. Mater. 2016, 28 (42), 9266–9291. 10.1002/adma.201602270. 27569575

[ref7] Suen N. T.; Hung S. F.; Quan Q.; Zhang N.; Xu Y. J.; Chen H. M. Electrocatalysis for the oxygen evolution reaction: recent development and future perspectives. Chem. Soc. Rev. 2017, 46 (2), 337–365. 10.1039/C6CS00328A. 28083578

[ref8] Song J.; Wei C.; Huang Z. F.; Liu C.; Zeng L.; Wang X.; Xu Z. J. A review on fundamentals for designing oxygen evolution electrocatalysts. Chem. Soc. Rev. 2020, 49 (7), 2196–2214. 10.1039/C9CS00607A. 32133479

[ref9] Yin J.; Jin J.; Lu M.; Huang B.; Zhang H.; Peng Y.; Xi P.; Yan C. H. Iridium Single Atoms Coupling with Oxygen Vacancies Boosts Oxygen Evolution Reaction in Acid Media. J. Am. Chem. Soc. 2020, 142 (43), 18378–18386. 10.1021/jacs.0c05050. 32955265

[ref10] Wu Z. Y.; Chen F. Y.; Li B.; Yu S. W.; Finfrock Y. Z.; Meira D. M.; Yan Q. Q.; Zhu P.; Chen M. X.; Song T. W.; et al. Non-iridium-based electrocatalyst for durable acidic oxygen evolution reaction in proton exchange membrane water electrolysis. Nat. Mater. 2023, 22 (1), 100–108. 10.1038/s41563-022-01380-5. 36266572

[ref11] Huynh M.; Shi C.; Billinge S. J.; Nocera D. G. Nature of Activated Manganese Oxide for Oxygen Evolution. J. Am. Chem. Soc. 2015, 137 (47), 14887–14904. 10.1021/jacs.5b06382. 26574923

[ref12] Huynh M.; Bediako D. K.; Nocera D. G. A functionally stable manganese oxide oxygen evolution catalyst in acid. J. Am. Chem. Soc. 2014, 136 (16), 6002–6010. 10.1021/ja413147e. 24669981

[ref13] Xiong Q.; Zhang X.; Wang H.; Liu G.; Wang G.; Zhang H.; Zhao H. One-step synthesis of cobalt-doped MoS_2_ nanosheets as bifunctional electrocatalysts for overall water splitting under both acidic and alkaline conditions. Chem. Commun. 2018, 54 (31), 3859–3862. 10.1039/C8CC00766G. 29594298

[ref14] Chong L.; Gao G.; Wen J.; Li H.; Xu H.; Green Z.; Sugar J. D.; Kropf A. J.; Xu W.; Lin X. M.; Xu H.; Wang L. W.; Liu D. J. La- and Mn-doped cobalt spinel oxygen evolution catalyst for proton exchange membrane electrolysis. Science 2023, 380, 609–616. 10.1126/science.ade1499. 37167381

[ref15] Yu J.; Garces-Pineda F. A.; Gonzalez-Cobos J.; Pena-Diaz M.; Rogero C.; Gimenez S.; Spadaro M. C.; Arbiol J.; Barja S.; Galan-Mascaros J. R. Sustainable oxygen evolution electrocatalysis in aqueous 1 M H_2_SO_4_ with earth abundant nanostructured Co_3_O_4_. Nat. Commun. 2022, 13 (1), 4341 10.1038/s41467-022-32024-6. 35896541 PMC9329283

[ref16] Huynh M.; Ozel T.; Liu C.; Lau E. C.; Nocera D. G. Design of template-stabilized active and earth-abundant oxygen evolution catalysts in acid. Chem. Sci. 2017, 8 (7), 4779–4794. 10.1039/C7SC01239J. 29163926 PMC5637126

[ref17] Burke M. S.; Kast M. G.; Trotochaud L.; Smith A. M.; Boettcher S. W. Cobalt-Iron (Oxy)hydroxide Oxygen Evolution Electrocatalysts: The Role of Structure and Composition on Activity, Stability, and Mechanism. J. Am. Chem. Soc. 2015, 137 (10), 3638–3648. 10.1021/jacs.5b00281. 25700234

[ref18] Smith R. D. L.; Prévot M. S.; Fagan R. D.; Zhang Z.; Sedach P. A.; Siu M. K. J.; Trudel S.; Berlinguette C. P. Photochemical Route for Accessing Amorphous Metal Oxide Materials for Water Oxidation Catalysis. Science 2013, 340 (6128), 60–63. 10.1126/science.1233638. 23539180

[ref19] Irani R.; Plate P.; Höhn C.; Bogdanoff P.; Wollgarten M.; Höflich K.; van de Krol R.; Abdi F. F. The Role of Ultra-thin MnO_x_ Co-Catalysts on the Photoelectrochemical Properties of BiVO_4_ Photoanodes. J. Mater. Chem. A 2020, 8 (11), 5508–5516. 10.1039/D0TA00939C.

[ref20] Lin F.; Boettcher S. W. Adaptive Semiconductor/Electrocatalyst Junctions in Water-Splitting Photoanodes. Nat. Mater. 2014, 13 (1), 81–86. 10.1038/nmat3811. 24292419

[ref21] Liu T.; Li W.; Wang D. Z.; Luo T.; Fei M.; Shin D.; Waegele M. M.; Wang D. Low Catalyst Loading Enhances Charge Accumulation for Photoelectrochemical Water Splitting. Angew. Chem., Int. Ed. Engl. 2023, 135 (34), e202307909 10.1002/ange.202307909. 37382150

[ref22] Guo Q.; Zhao Q.; Crespo-Otero R.; Di Tommaso D.; Tang J.; Dimitrov S. D.; Titirici M.-M.; Li X.; Jorge Sobrido A. B. Single-Atom Iridium on Hematite Photoanodes for Solar Water Splitting: Catalyst or Spectator?. J. Am. Chem. Soc. 2023, 145 (3), 1686–1695. 10.1021/jacs.2c09974. 36631927 PMC9880996

[ref23] Tilley S. D.; Cornuz M.; Sivula K.; Grätzel M. Light-Induced Water Splitting with Hematite: Improved Nanostructure and Iridium Oxide Catalysis. Angew. Chem., Int. Ed. Engl. 2010, 49 (36), 6405–6408. 10.1002/anie.201003110. 20665613

[ref24] Liu G.; Eichhorn J.; Jiang C.-M.; Scott M. C.; Hess L. H.; Gregoire J. M.; Haber J. A.; Sharp I. D.; Toma F. M. Interface Engineering for Light-Driven Water Oxidation: Unravelling the Passivating and Catalytic Mechanism in BiVO_4_ Overlayers. Sustainable Energy Fuels 2019, 3 (1), 127–135. 10.1039/C8SE00473K.

[ref25] Jang J.-W.; Du C.; Ye Y.; Lin Y.; Yao X.; Thorne J.; Liu E.; McMahon G.; Zhu J.; Javey A.; Guo J.; Wang D. Enabling Unassisted Solar Water Splitting by Iron Oxide and Silicon. Nat. Commun. 2015, 6 (1), 7447 10.1038/ncomms8447. 26078190 PMC4490416

[ref26] Thorne J. E.; Jang J.-W.; Liu E. Y.; Wang D. Understanding the Origin of Photoelectrode Performance Enhancement by Probing Surface Kinetics. Chem. Sci. 2016, 7 (5), 3347–3354. 10.1039/C5SC04519C. 29997828 PMC6006950

[ref27] Ye K.-H.; Wang Z.; Gu J.; Xiao S.; Yuan Y.; Zhu Y.; Zhang Y.; Mai W.; Yang S. Carbon Quantum Dots as a Visible Light Sensitizer to Significantly Increase the Solar Water Splitting Performance of Bismuth Vanadate Photoanodes. Energy Environ. Sci. 2017, 10 (3), 772–779. 10.1039/C6EE03442J.

[ref28] Kim T. W.; Choi K.-S. Nanoporous BiVO_4_ Photoanodes with Dual-Layer Oxygen Evolution Catalysts for Solar Water Splitting. Science 2014, 343 (6174), 990–994. 10.1126/science.1246913. 24526312

[ref29] Nellist M. R.; Laskowski F. A. L.; Qiu J.; Hajibabaei H.; Sivula K.; Hamann T. W.; Boettcher S. W. Potential-Sensing Electrochemical Atomic Force Microscopy for In Operando Analysis of Water-Splitting Catalysts and Interfaces. Nat. Energy 2018, 3 (1), 46–52. 10.1038/s41560-017-0048-1.

[ref30] Ma Y.; Kafizas A.; Pendlebury S. R.; Le Formal F.; Durrant J. R. Photoinduced Absorption Spectroscopy of CoPi on BiVO_4_: The Function of CoPi during Water Oxidation. Adv. Funct. Mater. 2016, 26 (27), 4951–4960. 10.1002/adfm.201600711.

[ref31] Ma Y.; Le Formal F.; Kafizas A.; Pendlebury S. R.; Durrant J. R. Efficient Suppression of Back Electron/Hole Recombination in Cobalt Phosphate Surface-Modified Undoped Bismuth Vanadate Photoanodes. J. Mater. Chem. A 2015, 3 (41), 20649–20657. 10.1039/C5TA05826K. PMC4894069 27358733

[ref32] Abdi F. F.; Firet N.; van de Krol R. Efficient BiVO_4_ Thin Film Photoanodes Modified with Cobalt Phosphate Catalyst and W-doping. ChemCatChem 2013, 5 (2), 490–496. 10.1002/cctc.201200472.

[ref33] Abdi F. F.; van de Krol R. Nature and Light Dependence of Bulk Recombination in Co-Pi-Catalyzed BiVO_4_ Photoanodes. J. Phys. Chem. C 2012, 116 (17), 9398–9404. 10.1021/jp3007552.

[ref34] Zhong D. K.; Choi S.; Gamelin D. R. Near-Complete Suppression of Surface Recombination in Solar Photoelectrolysis by “Co-Pi” Catalyst-Modified W:BiVO_4_. J. Am. Chem. Soc. 2011, 133 (45), 18370–18377. 10.1021/ja207348x. 21942320

[ref35] Klahr B.; Gimenez S.; Fabregat-Santiago F.; Bisquert J.; Hamann T. W. Photoelectrochemical and Impedance Spectroscopic Investigation of Water Oxidation with “Co-Pi”-Coated Hematite Electrodes. J. Am. Chem. Soc. 2012, 134 (40), 16693–16700. 10.1021/ja306427f. 22950478

[ref36] Barroso M.; Cowan A. J.; Pendlebury S. R.; Grätzel M.; Klug D. R.; Durrant J. R. The Role of Cobalt Phosphate in Enhancing the Photocatalytic Activity of α-Fe_2_O_3_ toward Water Oxidation. J. Am. Chem. Soc. 2011, 133 (38), 14868–14871. 10.1021/ja205325v. 21861508

[ref37] Zheng M.; Cao X.; Ding Y.; Tian T.; Lin J. Boosting Photocatalytic Water Oxidation Achieved by BiVO_4_ Coupled with Iron-Containing Polyoxometalate: Analysis the True Catalyst. J. Catal. 2018, 363, 109–116. 10.1016/j.jcat.2018.04.022.

[ref38] Lauinger S. M.; Piercy B. D.; Li W.; Yin Q.; Collins-Wildman D. L.; Glass E. N.; Losego M. D.; Wang D.; Geletii Y. V.; Hill C. L. Stabilization of Polyoxometalate Water Oxidation Catalysts on Hematite by Atomic Layer Deposition. ACS Appl. Mater. Interfaces 2017, 9 (40), 35048–35056. 10.1021/acsami.7b12168. 28929745

[ref39] Lauinger S. M.; Sumliner J. M.; Yin Q.; Xu Z.; Liang G.; Glass E. N.; Lian T.; Hill C. L. High Stability of Immobilized Polyoxometalates on TiO_2_ Nanoparticles and Nanoporous Films for Robust, Light-Induced Water Oxidation. Chem. Mater. 2015, 27 (17), 5886–5891. 10.1021/acs.chemmater.5b01248.

[ref40] Casadevall C. Heterogenization of Molecular Water Oxidation Catalysts in Electrodes for (Photo)Electrochemical Water Oxidation. Water 2022, 14 (3), 371 10.3390/w14030371.

[ref41] Wang M.; Yang Y.; Shen J.; Jiang J.; Sun L. Visible-Light-Absorbing Semiconductor/Molecular Catalyst Hybrid Photoelectrodes for H_2_ or O_2_ Evolution: Recent Advances and Challenges. Sustainable Energy Fuels 2017, 1 (8), 1641–1663. 10.1039/C7SE00222J.

[ref42] Yin Q.; Tan J. M.; Besson C.; Geletii Y. V.; Musaev D. G.; Kuznetsov A. E.; Luo Z.; Hardcastle K. I.; Hill C. L. A Fast Soluble Carbon-Free Molecular Water Oxidation Catalyst Based on Abundant Metals. Science 2010, 328, 342–345. 10.1126/science.1185372. 20223949

[ref43] Vickers J. W.; Lv H.; Sumliner J. M.; Zhu G.; Luo Z.; Musaev D. G.; Geletii Y. V.; Hill C. L. Differentiating homogeneous and heterogeneous water oxidation catalysis: confirmation that [Co_4_(H_2_O)_2_(α-PW_9_O_34_)_2_]^10–^ is a molecular water oxidation catalyst. J. Am. Chem. Soc. 2013, 135 (38), 14110–14118. 10.1021/ja4024868. 23977835

[ref44] Lv H.; Song J.; Geletii Y. V.; Vickers J. W.; Sumliner J. M.; Musaev D. G.; Kogerler P.; Zhuk P. F.; Bacsa J.; Zhu G.; Hill C. L. An exceptionally fast homogeneous carbon-free cobalt-based water oxidation catalyst. J. Am. Chem. Soc. 2014, 136 (26), 9268–9271. 10.1021/ja5045488. 24937166

[ref45] Blasco-Ahicart M.; Soriano-Lopez J.; Carbo J. J.; Poblet J. M.; Galan-Mascaros J. R. Polyoxometalate Electrocatalysts Based on Earth-Abundant Metals for Efficient Water Oxidation in Acidic Media. Nat. Chem. 2018, 10 (1), 24–30. 10.1038/nchem.2874. 29256497

[ref46] Arens J. T.; Blasco-Ahicart M.; Azmani K.; Soriano-López J.; García-Eguizábal A.; Poblet J. M.; Galan-Mascaros J. R. Water oxidation electrocatalysis in acidic media with Co-containing polyoxometalates. J. Catal. 2020, 389, 345–351. 10.1016/j.jcat.2020.06.006.

[ref47] Goberna-Ferron S.; Vigara L.; Soriano-Lopez J.; Galan-Mascaros J. R. Identification of a nonanuclear {Co^II9^} polyoxometalate cluster as a homogeneous catalyst for water oxidation. Inorg. Chem. 2012, 51 (21), 11707–11715. 10.1021/ic301618h. 23078372

[ref48] Mukhopadhyay S.; Debgupta J.; Singh C.; Kar A.; Das S. K. A Keggin Polyoxometalate Shows Water Oxidation Activity at Neutral pH: POM@ZIF-8, an Efficient and Robust Electrocatalyst. Angew. Chem., Int. Ed. Engl. 2018, 57 (7), 1918–1923. 10.1002/anie.201711920. 29240276

[ref49] Paille G.; Gomez-Mingot M.; Roch-Marchal C.; Lassalle-Kaiser B.; Mialane P.; Fontecave M.; Mellot-Draznieks C.; Dolbecq A. A Fully Noble Metal-Free Photosystem Based on Cobalt-Polyoxometalates Immobilized in a Porphyrinic Metal-Organic Framework for Water Oxidation. J. Am. Chem. Soc. 2018, 140 (10), 3613–3618. 10.1021/jacs.7b11788. 29393639

[ref50] Folkman S. J.; Soriano-Lopez J.; Galan-Mascaros J. R.; Finke R. G. Electrochemically Driven Water-Oxidation Catalysis Beginning with Six Exemplary Cobalt Polyoxometalates: Is It Molecular, Homogeneous Catalysis or Electrode-Bound, Heterogeneous CoO_x_ Catalysis?. J. Am. Chem. Soc. 2018, 140 (38), 12040–12055. 10.1021/jacs.8b06303. 30204436

[ref51] Sullivan K. P.; Wieliczko M.; Kim M.; Yin Q.; Collins-Wildman D. L.; Mehta A. K.; Bacsa J.; Lu X.; Geletii Y. V.; Hill C. L. Speciation and Dynamics in the [Co_4_V_2_W_18_O_68_]^10–^/Co(II)aq/CoO_x_ Catalytic Water Oxidation System. ACS Catal. 2018, 8 (12), 11952–11959. 10.1021/acscatal.7b01030.

[ref52] Huang Z.; Luo Z.; Geletii Y. V.; Vickers J. W.; Yin Q.; Wu D.; Hou Y.; Ding Y.; Song J.; Musaev D. G.; Hill C. L.; Lian T. Efficient light-driven carbon-free cobalt-based molecular catalyst for water oxidation. J. Am. Chem. Soc. 2011, 133 (7), 2068–2071. 10.1021/ja109681d. 21268644

[ref53] Martin-Sabi M.; Soriano-Lopez J.; Winter R. S.; Chen J. J.; Vila-Nadal L.; Long D. L.; Galan-Mascaros J. R.; Cronin L. Redox tuning the Weakley-type polyoxometalate archetype for the oxygen evolution reaction. Nat. Catal. 2018, 1 (3), 208–213. 10.1038/s41929-018-0037-1. 30079397 PMC6075698

[ref54] Wang Y.; Cao X.; Hu Q.; Liang X.; Tian T.; Lin J.; Yue M.; Ding Y. FeO_x_ Derived from an Iron-Containing Polyoxometalate Boosting the Photocatalytic Water Oxidation Activity of Ti^3+^-Doped TiO_2_. ACS Appl. Mater. Interfaces 2019, 11 (26), 23135–23143. 10.1021/acsami.9b03714. 31252488

[ref55] Hu Q.; Meng X.; Dong Y.; Han Q.; Wang Y.; Ding Y. A Stable Iron-Containing Polyoxometalate Coupled with Semiconductor for Efficient Photocatalytic Water Oxidation under Acidic Condition. Chem. Commun. 2019, 55 (78), 11778–11781. 10.1039/C9CC05726A. 31517345

[ref56] El-Deen S. S.; Hashem A. M.; Abdel Ghany A. E.; Indris S.; Ehrenberg H.; Mauger A.; Julien C. M. Anatase TiO2 nanoparticles for lithium-ion batteries. Ionics 2018, 24 (10), 2925–2934. 10.1007/s11581-017-2425-y.

[ref57] Challagulla S.; Tarafder K.; Ganesan R.; Roy S. Structure Sensitive Photocatalytic Reduction of Nitroarenes over TiO_2_. Sci. Rep. 2017, 7 (1), 8783 10.1038/s41598-017-08599-2. 28821751 PMC5562743

[ref58] Chen X.; Burda C. The Electronic Origin of the Visible-Light Absorption Properties of C-N- and S-Doped TiO_2_ Nanomaterials. J. Am. Chem. Soc. 2008, 130, 5018–5019. 10.1021/ja711023z. 18361492

[ref59] Amorello D.; Ledda F.; Romano V.; Zingales R. Batch Experiments for the Determination of the Ti(IV,III) Couple Formal Potential in 1 mol·dm^–3^ HCl, 2 mol·dm^–3^ NaCl Medium at 25 °C. J. Solution Chem. 2009, 38 (2), 259–263. 10.1007/s10953-008-9359-y.

[ref60] Wu H.; Zhang L.; Du A.; Irani R.; Van De Krol R.; Abdi F. F.; Ng Y. H. Low-Bias Photoelectrochemical Water Splitting via Mediating Trap States and Small Polaron Hopping. Nat. Commun. 2022, 13 (1), 6231 10.1038/s41467-022-33905-6. 36266344 PMC9585101

[ref61] Xing W.; Yin M.; Lv Q.; Hu Y.; Liu C.; Zhang J. 1—Oxygen Solubility, Diffusion Coefficient, and Solution Viscosity. Rotating Electrode Methods and Oxygen Reduction Electrocatalysts; Xing W., Yin G., Zhang J., Eds.; Elsevier, 2014; pp 1–31.

[ref62] Peter L. M.; Walker A. B.; Bein T.; Hufnagel A. G.; Kondofersky I. Interpretation of photocurrent transients at semiconductor electrodes: Effects of band-edge unpinning. J. Electroanal. Chem. 2020, 872, 114234 10.1016/j.jelechem.2020.114234.

[ref63] Yoshihara T.; Katoh R.; Furube A.; Tamaki Y.; Murai M.; Hara K.; Murata S.; Arakawa H.; Tachiya M. Identification of Reactive Species in Photoexcited Nanocrystalline TiO_2_ Films by Wide-Wavelength-Range (400–2500 nm) Transient Absorption Spectroscopy. J. Phys. Chem. B 2004, 108 (12), 3817–3823. 10.1021/jp031305d.

[ref64] Cowan A. J.; Tang J.; Leng W.; Durrant J. R.; Klug D. R. Water Splitting by Nanocrystalline TiO_2_ in a Complete Photoelectrochemical Cell Exhibits Efficiencies Limited by Charge Recombination. J. Phys. Chem. C 2010, 114 (9), 4208–4214. 10.1021/jp909993w.

[ref65] Kafizas A.; Wang X.; Pendlebury S. R.; Barnes P.; Ling M.; Sotelo-Vazquez C.; Quesada-Cabrera R.; Li C.; Parkin I. P.; Durrant J. R. Where Do Photogenerated Holes Go in Anatase:Rutile TiO_2_? A Transient Absorption Spectroscopy Study of Charge Transfer and Lifetime. J. Phys. Chem. A 2016, 120 (5), 715–723. 10.1021/acs.jpca.5b11567. 26777898

[ref66] Jing L.; Zhou J.; Durrant J. R.; Tang J.; Liu D.; Fu H. Dynamics of Photogenerated Charges in the Phosphate Modified TiO_2_ and the Enhanced Activity for Photoelectrochemical Water Splitting. Energy Environ. Sci. 2012, 5 (4), 6552–6558. 10.1039/c2ee03383f.

[ref67] Xiang X.; Fielden J.; Rodríguez-Córdoba W.; Huang Z.; Zhang N.; Luo Z.; Musaev D. G.; Lian T.; Hill C. L. Electron Transfer Dynamics in Semiconductor-Chromophore-Polyoxometalate Catalyst Photoanodes. J. Phys. Chem. C 2013, 117 (2), 918–926. 10.1021/jp312092u.

[ref68] Yang W.; Godin R.; Kasap H.; Moss B.; Dong Y.; Hillman S. A. J.; Steier L.; Reisner E.; Durrant J. R. Electron Accumulation Induces Efficiency Bottleneck for Hydrogen Production in Carbon Nitride Photocatalysts. J. Am. Chem. Soc. 2019, 141 (28), 11219–11229. 10.1021/jacs.9b04556. 31265274

[ref69] Sachs M.; Pastor E.; Kafizas A.; Durrant J. R. Evaluation of Surface State Mediated Charge Recombination in Anatase and Rutile TiO_2_. J. Phys. Chem. Lett. 2016, 7 (19), 3742–3746. 10.1021/acs.jpclett.6b01501. 27564137 PMC5056403

[ref70] Godin R.; Wang Y.; Zwijnenburg M. A.; Tang J.; Durrant J. R. Time-Resolved Spectroscopic Investigation of Charge Trapping in Carbon Nitrides Photocatalysts for Hydrogen Generation. J. Am. Chem. Soc. 2017, 139 (14), 5216–5224. 10.1021/jacs.7b01547. 28319382

[ref71] Nelson J.; Chandler R. E. Random Walk Models of Charge Transfer and Transport in Dye Sensitized Systems. Coord. Chem. Rev. 2004, 248 (13–14), 1181–1194. 10.1016/j.ccr.2004.04.001.

[ref72] Salvador P. Hole diffusion length in n-TiO_2_ single crystals and sintered electrodes: Photoelectrochemical determination and comparative analysis. J. Appl. Phys. 1984, 55 (8), 2977–2985. 10.1063/1.333358.

[ref73] Yamakata A.; Ishibashi T.-a.; Onishi H. Time-Resolved Infrared Absorption Spectroscopy of Photogenerated Electrons in Platinized TiO_2_ Particles. Chem. Phys. Lett. 2001, 333 (3–4), 271–277. 10.1016/S0009-2614(00)01374-9.

[ref74] Yamakata A.; Ishibashi T.-A.; Onishi H. Water- and Oxygen-Induced Decay Kinetics of Photogenerated Electrons in TiO_2_ and Pt/TiO_2_: A Time-Resolved Infrared Absorption Study. J. Phys. Chem. B 2001, 105 (30), 7258–7262. 10.1021/jp010802w.

[ref75] Song J.; Long J.; Liu Y.; Xu Z.; Ge A.; Piercy B. D.; Cullen D. A.; Ivanov I. N.; McBride J. R.; Losego M. D.; Lian T. Highly Efficient Plasmon Induced Hot-Electron Transfer at Ag/TiO_2_ Interface. ACS Photonics 2021, 8 (5), 1497–1504. 10.1021/acsphotonics.1c00321.

[ref76] Brito R.; Rodríguez V.; Figueroa J.; Cabrera C. R. Adsorption of 3-mercaptopropyltrimethoxysilane and 3-aminopropyltrimethoxysilane at Platinum Electrodes. J. Electroanal. Chem. 2002, 520 (1–2), 47–52. 10.1016/S0022-0728(01)00718-5.

[ref77] Ueda T. Electrochemistry of Polyoxometalates: From Fundamental Aspects to Applications. ChemElectroChem 2018, 5 (6), 823–838. 10.1002/celc.201701170.

[ref78] Kumar A.; Santangelo P. G.; Lewis N. S. Electrolysis of Water at Strontium Titanate (SrTiO3) Photoelectrodes: Distinguishing between the Statistical and Stochastic Formalisms for Electron-Transfer Processes in Fuel-Forming Photoelectrochemical Systems. J. Phys. Chem. 1992, 96 (2), 834–842. 10.1021/j100181a057.

[ref79] Iqbal A.; Bevan K. H. Simultaneously Solving the Photovoltage and Photocurrent at Semiconductor-Liquid Interfaces. J. Phys. Chem. C 2018, 122 (1), 30–43. 10.1021/acs.jpcc.7b08517.

