# Supplementary material for: Charge Transfer Mechanism on a Cobalt-Polyoxometalate-TiO2 Photoanode for Water Oxidation in Acid
Source: J Am Chem Soc. 2024 May 15;146(21):14600–9. doi: 10.1021/jacs.4c01441 (PMC11140742; doi:10.1021/jacs.4c01441)
Supplement: Supplementary file 1 — ja4c01441_si_001.pdf [file ja4c01441_si_001.pdf]

Supporting Information.

The charge transfer mechanism on a cobalt-polyoxometalate-TiO_2 photoanode for water oxidation in acid

Fengyi Zhao[a]†, Ting Cheng[a]†, Xinlin Lu[a], Nandan Ghorai[a], Yiwei Yang[a], Yurii V. Geletii[a], Djamaladdin G. Musaev[a,b], Craig L. Hill[a]*, Tianquan Lian[a]**

[a]Department of Chemistry, Emory University, Atlanta, GA, 30322, USA

[b]Cherry L. Emerson Centre for Scientific Computation, Emory University, 1515 Dickey Drive, Atlanta, GA, 30322, USA

† These authors contributed equally to this work.

*To whom correspondence should be addressed: tlian@emory.edu; chill@emory.edu; fzhao36@emroy.edu

Table of Contents

Materials 2

General Characterization Methods 3

Photoelectrochemical Characterization Methods 4

Transient Absorption (TA) Spectroscopy Methods..5

General Characterization 7

Control PEC Characterization and AC Impedance Fitting Parameters 10

Visible and IR Transient Absorption Spectroscopy 12

Materials

Materials and solvents were purchased as ACS analytical or reagent grade and used as received. $Na_8K_8[Co_9(H_2O)_6(OH)_3(HPO_4)_2(PW_9O_{34})_3]\cdot 49H_2O$ (**Co₉POM**) was obtained following a literature method. [1]

The preparation of TiO_2 and TiO_2 coated with **Co_9POM** photoelectrodes is based on our previous report with modifications. [2] The fluorine-doped tin oxide substrates, FTO (Pilkington TEC15, ~ 15 Ω/sq resistance), were cleaned by sonicating sequentially in deionized water, acetone, and ethanol for 20 min each time, followed by blowing dry with nitrogen gas. Before coating, the top area of substrate was covered by one layer of adhesive tape (40 µm thickness) to provide a noncoated area for electrical conductance for the final electrode. The bottom area (smaller than the top area) was also covered by one layer of adhesive tape for the following Doctor blade coating process. The 20 nm-sized TiO_2 paste (Dyesol, 90-T) was then applied in the center of the substrate and distributed with a glass rod sliding over the tape-covered substrates on the side. The substrates were sintered at 500 °C for 1 hour, yielding a ~10-20 µm thick nanostructured TiO_2 film. For TiO_2-APS sample, TiO_2 electrodes with the noncoated area covered by Teflon tape were suspended in an anhydrous toluene mixture of 6% 3-aminopropyltrimethoxysilane (APS) for 6 hours at 70 °C. After attachment of APS ligand, the resulting films were washed with toluene, acetone, and ethanol. The film was further soaked in DI water overnight to remove the excess APS ligand. For TiO_2-**Co_9POM** sample, the TiO_2-APS sample was soaked in a 1 mM aqueous solution of **Co_9POM** overnight then washed with toluene, acetone, and ethanol. The electrodes were rinsed with 5 mL of water three times and dried in air. Functionalization of TiO_2 with $H_3PW_{12}O_4$ (TiO_2-$H_3PW_{12}O_{40}$) followed the same procedure for making TiO_2-**Co_9POM**, the only difference was soaking TiO_2-APS in 1mM $H_3PW_{12}O_{40}$ rather than in 1mM of **Co_9POM** overnight. For all photoanode samples,

the top area of the electrodes was connected with a conductive copper tape and sealed with Epoxy adhesive (Henkel Loctite Hysol 1C Epoxi).

TiO_2 photoelectrode modified with the cobalt-phosphate water oxidation catalyst (Co-Pi) [3] was prepared following the literature procedure. [4, 5] A three-electrode cell was used with TiO_2 working electrode, Ag/AgCl (1 M KCl) reference electrode, and Pt mesh counter electrode. A potential of 0.9 V vs. Ag/AgCl was applied on TiO_2 working electrode immersed in a solution of 0.5 mM cobalt nitrate with 0.1 M potassium phosphate buffer at pH 7 under 365 nm CW illumination. Co–Pi is photoelectrochemically deposited on TiO_2 by passing 1 cm^2 electrode with 2.4 mC of current.

General Characterization Methods

The purity of the **Co_9POM** was confirmed by the Fourier transform infrared (FT-IR) (Figure S1a) and the water content was determined by thermogravimetric analysis (TGA) (Figure S1b). Infrared spectra (2% sample in KBr pellet) were recorded on a Nicolet TM 6700 Fourier transform infrared (FT-IR) spectrometer. X-ray photoelectron spectroscopy (XPS) was conducted on a thermos K-ALPHA XPS instrument. The TGA data were collected on a Mettler Toledo TGA instrument. Diffuse reflectance spectroscopy was measured on a Cary 5000 UV-Vis/NIR instrument. Gas chromatography (GC) of the gas phase was analyzed using an Agilent 7890 gas chromatograph with a 5 Å molecular sieve column, a thermal conductivity detector, and argon as carrier gas. The FOXY Forspor probe experiments are carried out using a NeoFox Phase Measurement system (Ocean Insight, Orlando, USA) equipped with FOSPOR-R oxygen sensor (1000-micron fiber). The Raman spectroscopy was conducted using a home-built Raman setup as described in previous literature. [6] ICP data were collected using a Thermo Fisher iCAP ICP-MS (Inductively Coupled Plasma - Mass Spectrometer) instrument.

Photoelectrochemical Characterization Methods

Cyclic voltammograms (CVs), linear sweep voltammograms (LSVs) and bulk electrolysis data were obtained using a BAS CV-50W electrochemical analyzer and conducted at room temperature (25 ± 2 °C) in a custom 50 mL quartz cell (C012-2, Gaoss Union) equipped with airtight adapters and purged with argon gas prior to use. Mott-Schottky and AC impedance measurements are performed with a CHI 660e electrochemical station in the same quartz cell.

In Mott-Schottky experiment, 500 Hz of 5 mV of AC voltage were used in superposition with the DC potential. In AC impedance measurements, the potential is held at 1.23 V_{RHE}, with 5 mV of AC voltage scanning frequency from 100 kHz to 1 Hz.

CVs and LSVs were recorded in a standard three-electrode configuration with a glassy carbon working electrode (S = 0.07 cm^2) or a **Co_9POM**-modified working electrode using FTO as conductive substrate (S = 1.0 cm^2), a platinum wire counter electrode, and a 3 M KCl Ag/AgCl (+0.210 V vs. RHE) reference electrode in a 0.1 M pH = 2 sulfate buffer solution as determined by an Orion pH meter (model 230A). In this work, all potentials measured against Ag/AgCl were then converted to the reversible hydrogen electrode (RHE) scale using: *E* (potential, versus RHE) = *E* (versus Ag/AgCl) + 0.210 V + 0.0591×pH. The scan rate used in voltametric experiments was 100 mV/s, and the LSVs were scanned in a positive direction. A 100 mW/cm^2 365 nm LED light was focused on the photoelectrode.

Bulk electrolysis was conducted in a 0.1 M pH = 2 sulfate buffer solution with the potential held at 0.73 V_{RHE} under 20 mW/cm^2 365 nm LED UV illumination. A gas-tight H-cell (C012-1, Gaoss Union) with a Nafion 115 proton exchange membrane is used for bulk electrolysis experiment. A TiO_2-**Co_9POM** photoanode working electrode and a Ag/AgCl reference electrode were in the anode chamber, and a platinum mesh counter electrode is in the cathode chamber. The

headspace during bulk electrolysis was then analyzed by GC as described in the method section above. The number of electrons transferred during bulk electrolysis was calculated by Faraday's law of electrolysis Q = nFN, where Q is the number of coulombs, F = 96485 C/mol is Faraday's constant, N is the moles of substrate electrolyzed and n is the stoichiometric number of electrons consumed (4 in the case of oxygen evolution reaction). In the GC measurements, when the data collection was complete, 1.7 C of charge had passed through the system. The headspace gas (200 μL) was injected into the GC for analysis and repeated 4 times to reduce the error.

Incident photon-to-current efficiencies (IPCEs) of TiO_2 and TiO_2-**Co_9POM** were measured under chopped light illumination at 1.23 V_{RHE} applied potential and calculated using the following equation:

$$\mathrm{IPCE} = \frac{J_{ph} \times 1239.8}{P \times \lambda} \times 100\%$$

where J_{ph} (mA/cm^2) is the photocurrent; P (mW/cm^2) is the power density of monochromatic incident light; λ (nm) is the wavelength of monochromatic light; and 1239.8 (V nm) is a coefficient from the multiplication of speed of light, reciprocal of unit charge and Planck's constant.

Transient Absorption (TA) Spectroscopy Methods

Both visible TA and mid-IR TA spectroscopy pump probe pulses are derived from an amplified Ti:sapphire laser system (Coherent Astrella, 800 nm, 5 mJ/pulse, 35 fs, and 1 kHz repetition rate).

In the TA-vis experiments, 40% of 800 nm fundamental output (2 mJ) was used to pump a visible Optical Parametric Amplifier (OPA) (Opera, Coherent) to generate a visible-light pump in TA-vis experiments. In the visible OPA, two tunable near-IR pulses, signal and idler, from 1.1 to 2.5 μm are generated. Signal and idler beams were separated with a dichroic mirror to generate

a tunable visible pump beam by sum-frequency generation with 800 nm or second-harmonic generation using a BBO crystal. In our study, a 350-nm pump beam was produced by fourth-harmonic generation of a 1400 nm signal beam. A continuously variable neutral-density filter wheel was used to adjust the power of the pump beam. The pump beam was focused on the sample with a beam waist of about 420 μm. Ten percent of the 5mJ/pulse output is used to generate a white light continuum (WLC) from 380 to 700 nm on a single crystal of CaF_2 as a probe in the TA-vis experiments. The WLC was split into probe and reference beams. The probe beam was focused with an aluminum parabolic mirror on the sample with a beam waist of 150 μm. After passing through the sample, the probe beam was focused on a fiber-coupled multichannel spectrometer with complementary metal-oxide-semiconductor (CMOS) sensors and detected at a frequency of 1 kHz. The delay between the pump and probe pulses was controlled by a motorized probe delay stage. The pump beam was chopped by a synchronized chopper to 500 Hz. The change in absorbance of the samples with and without pump was calculated. The samples used in the TA-vis experiments are deposited on FTO glass. The instrument response function (IRF) of this system was determined to be ~300 fs by measuring the pump-probe response on an FTO substrate.

In the TA-IR experiments, output from the same visible OPA was used as the pump beam; it was focused on the IR sample stage with a beam waist of 410 μm. 40 % of 800 nm fundamental out (2 mJ) was used to pump an IR OPA (Opera, Coherent). Two tunable near-IR signal and idler pulses from 1.1 to 2.5 μm were generated. The signal and idler pulses were further combined in a 1-mm-thick $AgGaS_2$ crystal to generate a tunable mid-IR pulse from 3 to 10 μm by difference frequency generation (DFG). The mid-IR beam was focused on the sample stage at a beam waist of around 290 μm to overlap with the pump beam. After passing the sample, mid-IR probe was dispersed into a spectrograph and detected with a 32-element infrared HgCdTe (MCT) array

detector at a frequency of 1k Hz. The pump beam was chopped by a synchronized chopper to 500 Hz. The typical IRF of TA-IR experiments was determined to be around 170 fs using a silicon wafer. The samples used in TA-IR experiments were deposited on a sapphire window to avoid strong IR absorption of FTO.

General Characterization

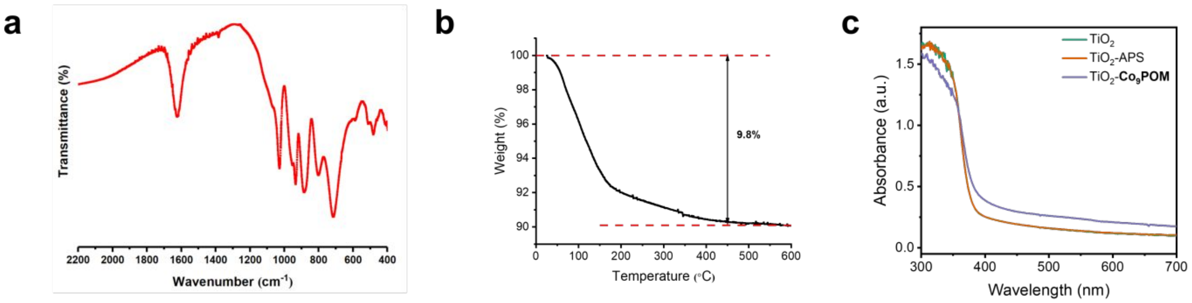

Figure S1. (a) FT-IR spectroscopy and (b) TGA of solid **Co_9POM**. (c) Diffuse-reflectance of TiO_2, TiO_2-APS, and TiO_2-**Co_9POM** samples on sapphire substrates.

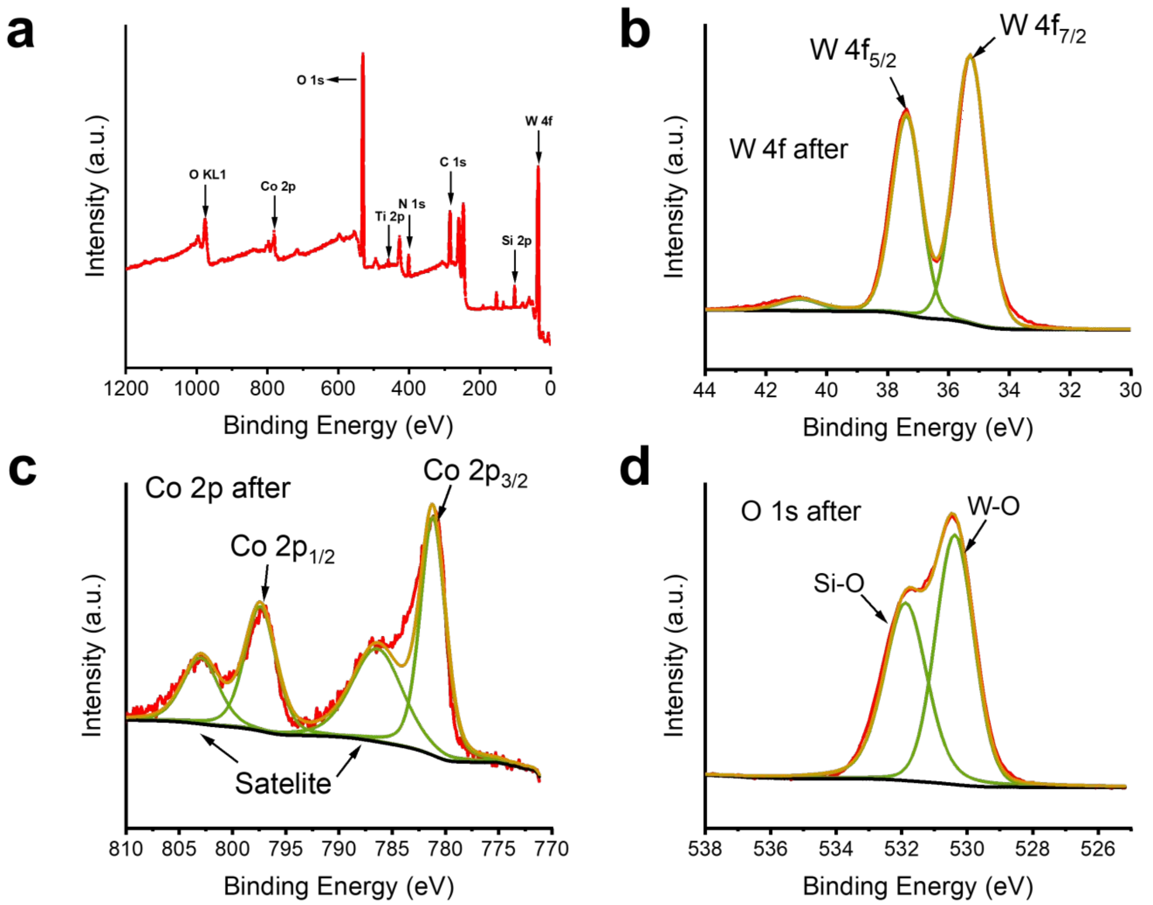

Figure S2. (a) Full XPS of TiO_2-**Co₉POM** after the bulk electrolysis. XPS of (b) W 4f, (c) Co 2p, and (d) O 1s of TiO_2-**Co_9POM** after bulk electrolysis.

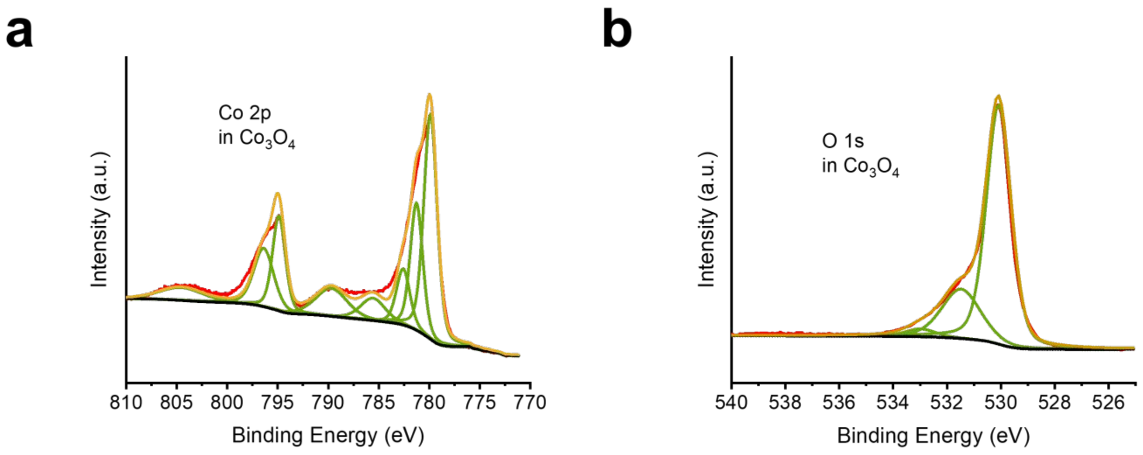

Figure S3. XPS of (a) Co 2p and (b) O 1s in Co_3O_4 solid.

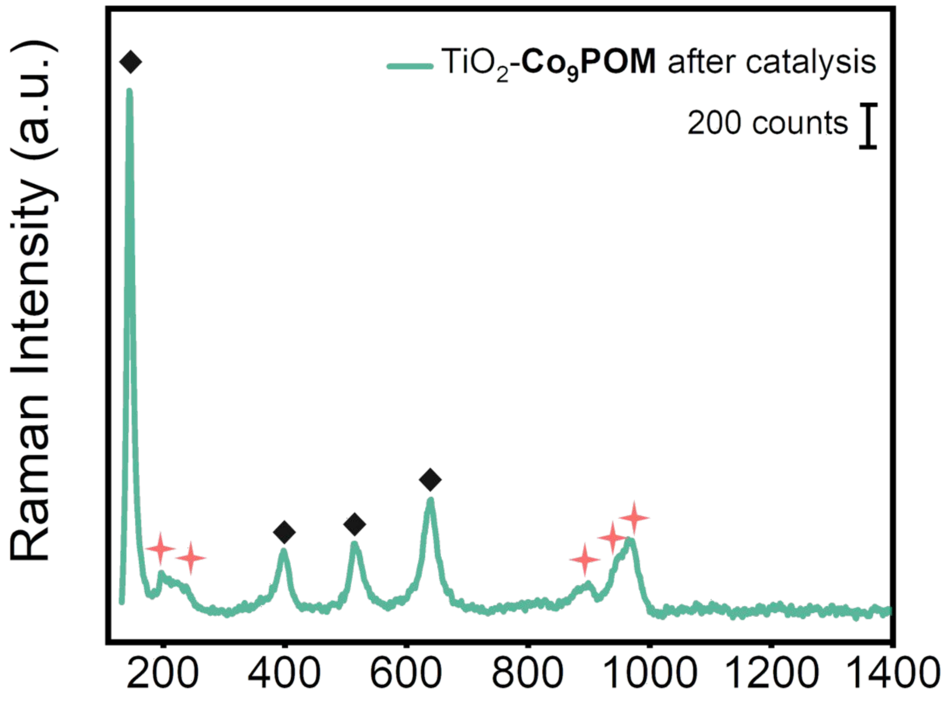

Figure S4. Raman spectroscopy of TiO_2-**Co_9POM** on FTO substrates after passing around 3.0 C of charge on the electrode for OER reaction as described in Figure 4b. Black diamonds and red stars represent the characteristic Raman peaks for anatase TiO_2 and **Co_9POM**, respectively.

Control PEC Characterization and AC Impedance Fitting Parameters

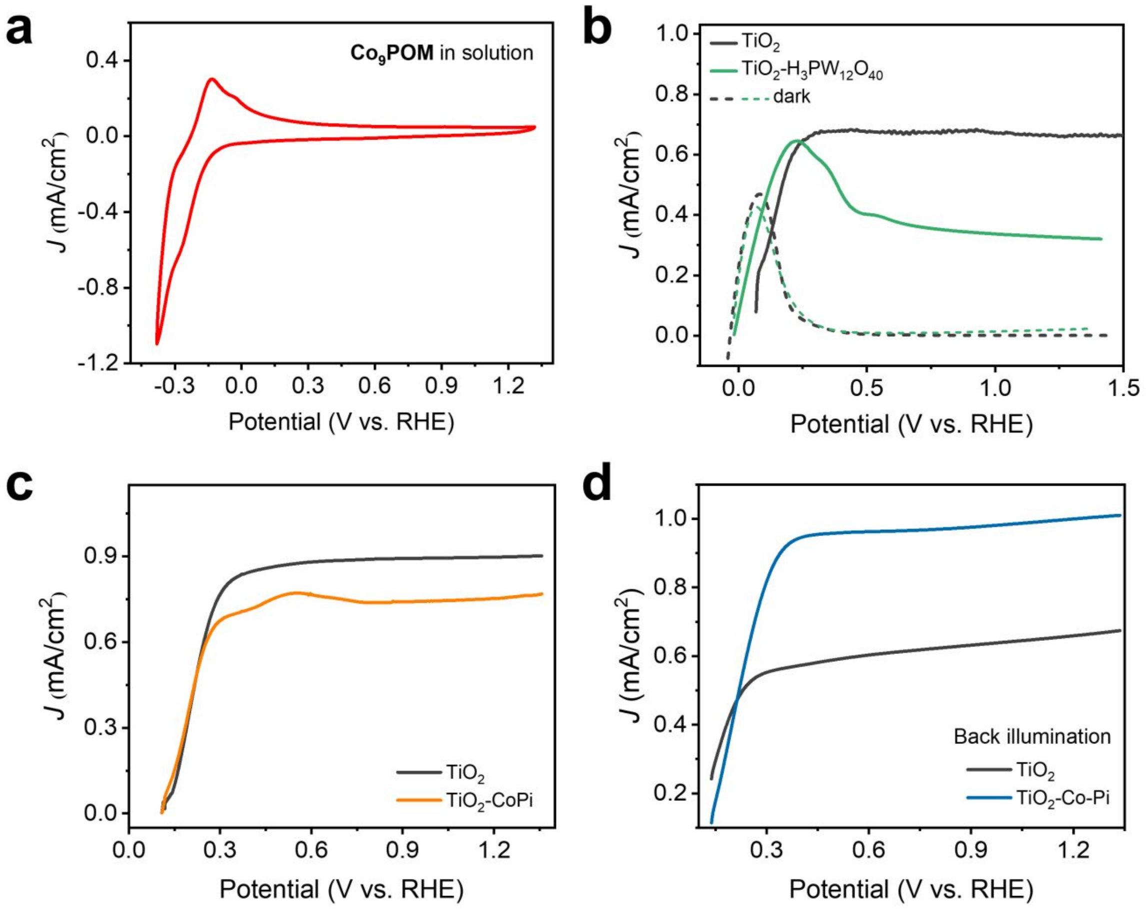

Figure S5. (a) Cyclic voltammetry of **Co_9POM** in solution; (b) J-V curve of $H_3PW_{12}O_{40}$-modified and bare TiO_2 photoelectrodes; and J-V photocurrent of Co-Pi-modified TiO_2 under 100 mW cm^{-2} 365-nm LED light illumination from (c) front illumination and (d) back illumination. A sulfate buffered solution at pH 2.0 served as the electrolyte.

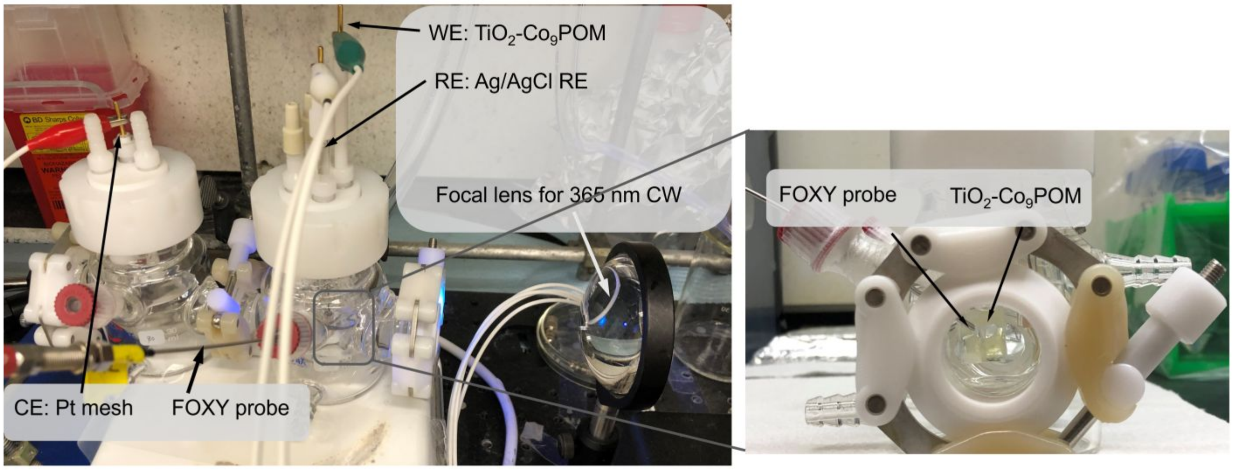

Figure S6. FOXY Forspor oxygen probe setup for measuring O_2 under OER conditions. The right panel is an enlargement showing the relative positions of the probe and electrode.

Table S1. AC impedance fitting parameters of TiO_2, TiO_2-APS and TiO_2-**Co_9POM** photoanodes.

	TiO_2	TiO_2-APS	TiO_2-**Co_9POM**
U_{fb} (V_{RHE})	-0.083 $\pm$ 0.017	-0.062 $\pm$ 0.020	-0.164 $\pm$ 0.009
R_s (Ohm/cm^2)	25.41 $\pm$ 0.17	27.88 $\pm$ 0.12	46.32 $\pm$ 0.28
R_{ct} (kOhm/cm^2)	567.8 $\pm$ 292	410.3 $\pm$ 80.6	153.3 $\pm$ 28.2
CPE-Q (μF/cm^2)	7.70 $\pm$ 0.10	6.67 $\pm$ 0.05	126.2 $\pm$ 0.16
CPE-α	0.978 $\pm$ 0.002	0.979 $\pm$ 0.001	0.904 $\pm$ 0.002

Visible and IR Transient Absorption Spectroscopy

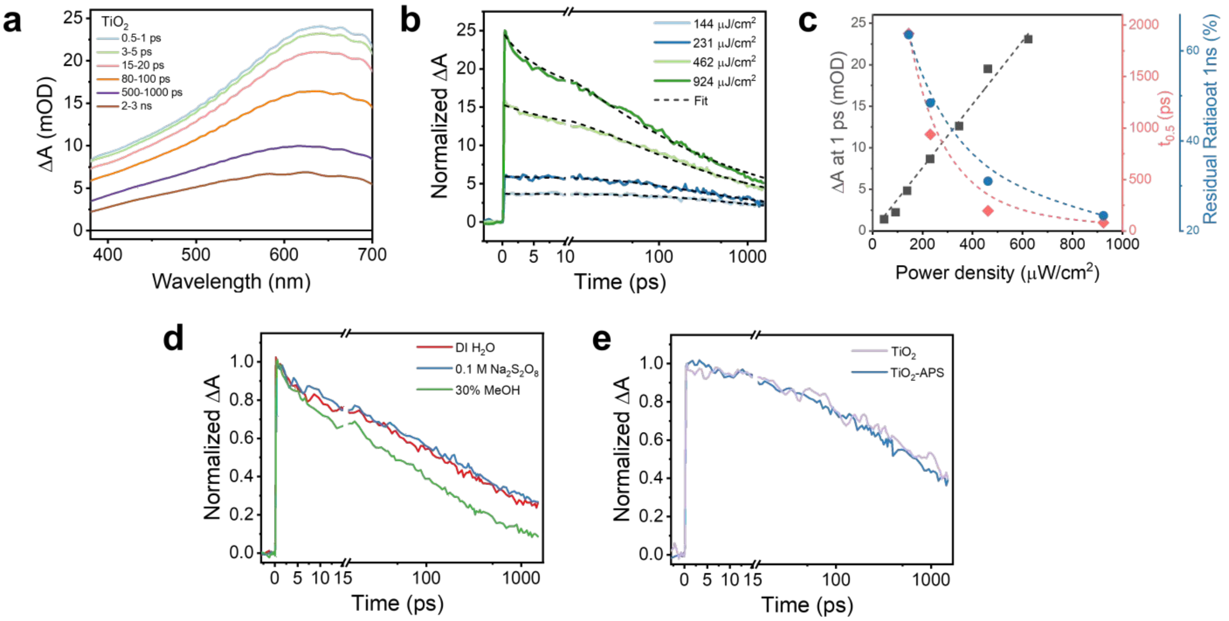

Figure S7. Transient absorption spectroscopy of TiO_2 on FTO pumped by a 350 nm pulses and probed by visible white light: (a) TA-vis spectra; excitation fluence dependence of (b) kinetic decay and (c) maximum signal amplitude, half lifetime and signal residual as a function of power density; (d) kinetic decay in DI water, 0.1 M $Na_2S_2O_8$, and 30% methanol; (e) normalized hole kinetic decay of TiO_2 and TiO_2-APS tested in air. All signal kinetics decays are extracted by averaging 460-520 nm; the excitation power density of 462 μJ/cm^2 is used in (a) and 231 μJ/cm^2 is used in (d) and (e).

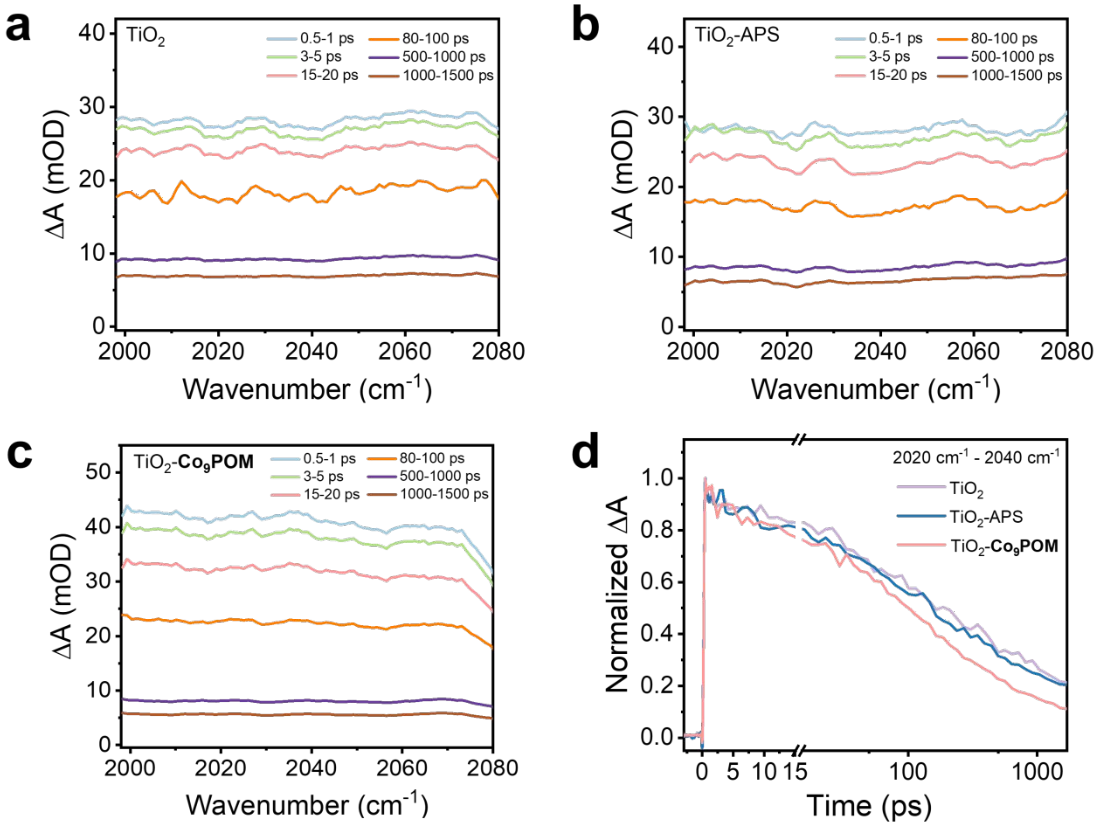

Figure S8. TA-IR spectra of (a) TiO_2, (b) TiO_2-APS, and (c) TiO_2-**Co_9POM** samples. (d) Kinetics comparison of three samples from 2020-2040 cm^{-1}. TA-IR experiments are conducted using 462 μJ/cm^2 350-nm pump, with all samples deposited on sapphire windows.

References:

(1) Galán-Mascarós, J. R.; Gómez-Garcia, C. J.; Borrás-Almenar, J. J.; Coronado, E. High nuclearity magnetic clusters: Magnetic properties of a nine cobalt cluster encapsulated in a polyoxometalate, $[Co_9(OH)_3(H_2O)_6(HPO_4)_2(PW_9O_{34})_3]^{16-}$. *Adv. Mater.* **1994**, *6* (3), 221-223.
(2) Lauinger, S. M.; Sumliner, J. M.; Yin, Q.; Xu, Z.; Liang, G.; Glass, E. N.; Lian, T.; Hill, C. L. High Stability of Immobilized Polyoxometalates on TiO_2 Nanoparticles and Nanoporous Films for Robust, Light-Induced Water Oxidation. *Chem. Mater.* **2015**, *27* (17), 5886-5891.
(3) Kanan, M. W.; Nocera, D. G. In Situ Formation of an Oxygen-Evolving Catalyst in Neutral Water Containing Phosphate and Co^{2+}. *Science* **2008**, *321*, 1072-1075.
(4) Zhong, D. K.; Choi, S.; Gamelin, D. R. Near-complete suppression of surface recombination in solar photoelectrolysis by "Co-Pi" catalyst-modified W:$BiVO_4$. *J. Am. Chem. Soc.* **2011**, *133* (45), 18370-18377.
(5) Zhong, D. K.; Cornuz, M.; Sivula, K.; Grätzel, M.; Gamelin, D. R. Photo-assisted electrodeposition of cobalt–phosphate (Co–Pi) catalyst on hematite photoanodes for solar water oxidation. *Energy Environ. Sci.* **2011**, *4* (5), 1759-1764.
(6) Suo, S.; Sheehan, C.; Zhao, F.; Xiao, L.; Xu, Z.; Meng, J.; Mallouk, T. E.; Lian, T. Direct Vibrational Stark Shift Probe of Quasi-Fermi Level Alignment in Metal Nanoparticle Catalyst-Based Metal–Insulator–Semiconductor Junction Photoelectrodes. *J. Am. Chem. Soc.* **2023**.
